# Transcription factor CBF-1 is critical for circadian gene expression by modulating WHITE COLLAR complex recruitment to the *frq* locus

**DOI:** 10.1371/journal.pgen.1007570

**Published:** 2018-09-12

**Authors:** Xuemei Cao, Xiao Liu, Hongda Li, Yumeng Fan, Jiabin Duan, Yi Liu, Qun He

**Affiliations:** 1 State Key Laboratory of Agrobiotechnology and MOA Key Laboratory of Soil Microbiology, College of Biological Sciences, China Agricultural University, Beijing, China; 2 Department of Physiology, University of Texas Southwestern Medical Center, Dallas, Texas, United States of America; The University of North Carolina at Chapel Hill, UNITED STATES

## Abstract

Transcription of the *Neurospora crassa* circadian clock gene *frequency* (*frq*) is an essential process in the negative feedback loop that controls circadian rhythms. WHITE COLLAR 1 (WC-1) and WHITE COLLAR 2 (WC-2) forms the WC complex (WCC) that is the main activator of *frq* transcription by binding to its promoter. Here, we show that Centromere Binding Factor 1 (CBF-1) is a critical component of the *N*. *crassa* circadian clock by regulating *frq* transcription. Deletion of *cbf-1* resulted in long period and low amplitude rhythms, whereas overexpression of CBF-1 abolished the circadian rhythms. Loss of CBF-1 resulted in WC-independent FRQ expression and suppression of WCC activity. As WCC, CBF-1 also binds to the C-box at the *frq* promoter. Overexpression of CBF-1 impaired WCC binding to the C-box to suppress *frq* transcription. Together, our results suggest that the proper level of CBF-1 is critical for circadian clock function by suppressing WC-independent FRQ expression and by regulating WCC binding to the *frq* promoter.

## Author summary

Circadian clocks, which measure time on a scale of approximately 24 hours, are generated by a cell-autonomous circadian oscillator comprised of autoregulatory feedback loops. In the *Neurospora crassa* circadian oscillator, WHITE COLLAR complex (WCC) actives transcription of the *frequency* (*frq*) gene. FRQ inhibits the activity of WCC to close the negative feedback loop. Here, we showed that the transcription factor CBF-1 functions as a repressor to modulate WCC recruitment to the C-box of *frq* promoter. Our data showed that deletion or overexpression of CBF-1 dampened circadian rhythm due to impaired WCC binding at the *frq* promoter. As CBF-1 is conserved in eukaryotes, our data provide novel insights into the negative feedback mechanism that controls the biological clocks in other organisms.

## Introduction

Circadian rhythms exist in almost all kingdoms of life [1–3]. The circadian clock is a highly conserved timekeeping system that allows organisms to anticipate and adjust to daily environmental changes [4]. These rhythms are generated by self-sustained molecular oscillators, based on transcription-translation negative feedback loops in eukaryotic model organisms [1]. In *Neurospora crassa*, the loops involve in a number of core clock proteins including WHITE COLLAR proteins WC-1 and WC-2, FREQUENCY (FRQ), FRQ-interacting RNA helicase (FRH), Casein Kinase I (CKI), Casein Kinase II (CKII), and several other accessory factors [5–8].

In the *N*. *crassa* circadian system, WC-1 and WC-2, two PER-ARNT-SIM (PAS) domain-containing transcription factors, form a heterodimeric complex (WCC) that initiates transcription of the clock gene *frq* through binding to PLRE and C-box regulatory sequences [9, 10]. FRQ protein dimerizes and forms a complex with FRH, functioning as the negative element in the circadian negative-feedback loop [11]. Once FRQ protein is made, it becomes progressively phosphorylated. Then, the FRQ-FRH complex in conjunction with kinases, such as CKI and CKII, promotes phosphorylation of WCC, leading to its inactivation [11–18]. The phosphorylation kinetics determines circadian period length [19]. When FRQ protein becomes extensively phosphorylated, it is ubiquitinated by an E3 ligase complex and degraded through the proteasome, allowing the cycle to restart [19–22].

Rhythmic activation and repression of *frq* transcription generates rhythmic *frq* mRNA, which is the basis of circadian gene expression. Activation of the *frq* transcription by WCC has been characterized in detail [9, 10, 23]. Two WCC-binding elements within the promoter of *frq* are necessary to maximally induce *frq* transcription in response to light [10]. A distal element, the C-box, is necessary and sufficient to promote rhythmic *frq* expression in constant darkness (DD) [24, 25]. WC complex was long thought to be the only transcriptional activator of *frq* transcription [9]. However, we recently discovered that WC-independent *frq* transcription can be regulated by the RCO-1/RCM-1 complex, the SET-2 pathway, and the IEC-1-INO80 complex [26–29], suggesting that regulation of *frq* transcription is complex.

Centromere Binding Factor 1 (CBF-1) is initially identified by its ability to bind to centromeric DNA element I (CDEI) in *Saccharomyces cerevisiae* to ensure correct separation of chromosome [30]. CBF-1, which is a basic helix-loop-helix-leucine zipper (bHLH-LZ) factor, recognizes the consensus sequence 5’-CACGTG-3’ [31] and plays a key role in the regulation of methionine metabolism involving in the formation of the CBF-l-Met4-Met28 complex [32–34]. In phosphate metabolism, CBF-1 and the transcription factor Pho4 regulate the sensitivity of promoters to phosphate-concentration levels [35]. CBF-1 functions to modulate chromatin structure at centromeres and regulates transcription from several CDEI-carrying promoters (i.e., *MET25*, *TRP1*, *GAL2*) [36, 37]. Thus, CBF-1 is a multifunctional protein that influences a number of biological processes.

In this study, we discovered that the CBF-1 homolog in *N*. *crassa* is necessary for the normal function of circadian clock. Both overt and molecular rhythmicities were severely dampened in *cbf-1* deletion or overexpression strains. We found that CBF-1 rhythmically binds to the C-box of the *frq* promoter. Furthermore, loss of CBF-1 led to WC-independent *frq* transcription by decreasing RCM-1 recruitment to the *frq* locus. However, high levels of CBF-1 suppressed *frq* transcription by impairing WCC binding to the C-box, indicating that CBF-1 plays a critical role in regulating rhythmic WC-dependent *frq* transcription.

## Results

### Deletion of the *cbf-1* gene results in long period and low amplitude of circadian rhythms

**Fig 1:**
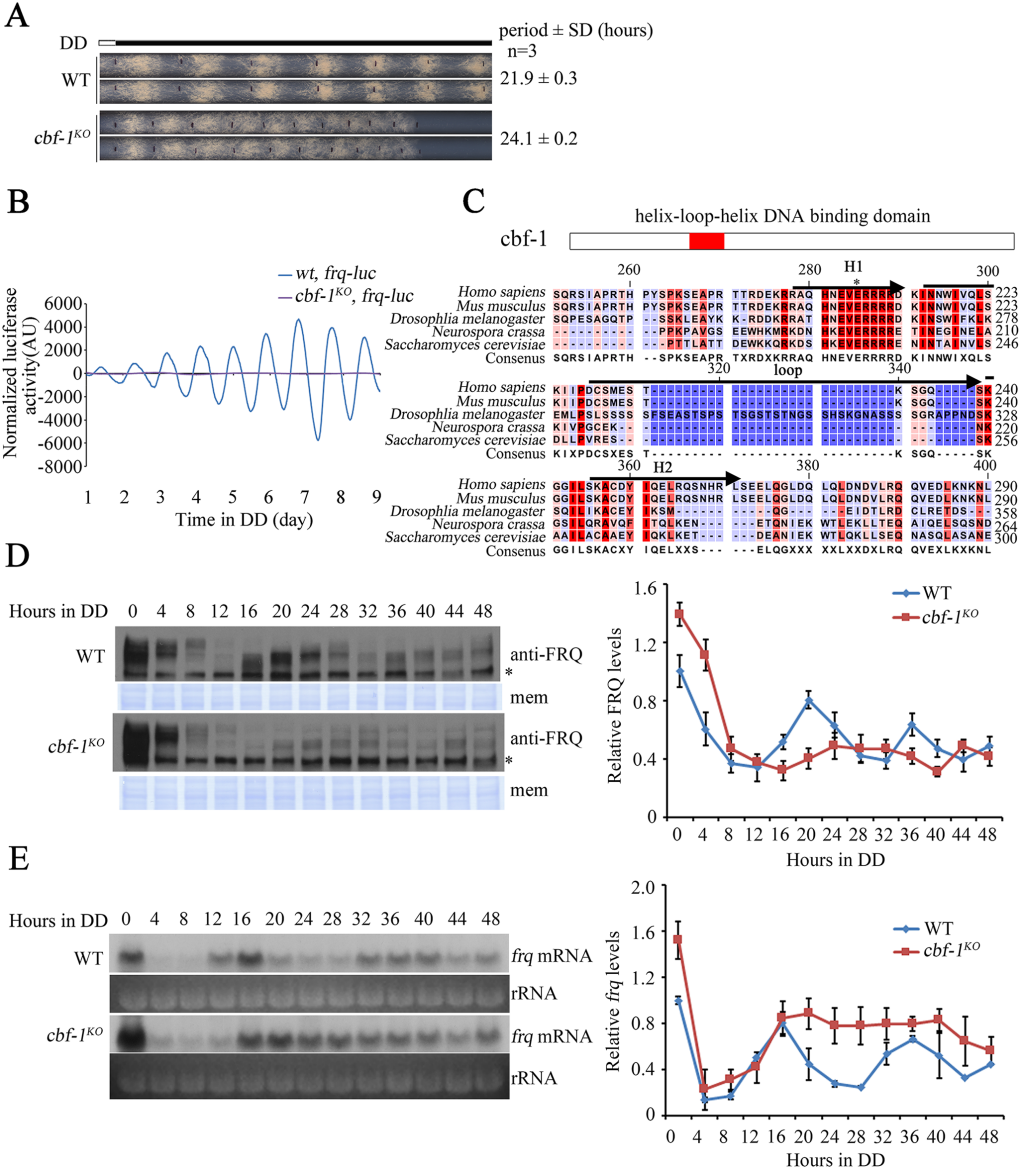
Deletion of the *cbf-1* gene results in long period and low amplitude of circadian rhythms. (A) Race tube analyses of the *cbf-1*^*KO*^ strains. (B) Luciferase activity of *wt*, *frq-luc* and *cbf-1*^*KO*^, *frq-luc* strains. The low normalized luciferase signal levels in the *cbf-1*^*KO*^, *frq-luc* strain reflect the low-amplitude fluctuation of luciferase activity. Raw data were normalized to subtract the baseline calculated by LumiCycle analysis software. (C) Amino acid sequence alignment of the HLH domain from the *Neurospora* CBF-1, *Saccharomyces* CBF-1, *Drosophila* USF1, mouse USF1, and human USF1. The HLH domain is composed of H_1_, Loop and H_2_. The asterisk indicates the conserved Glu (E9) in the basic domains of HLH proteins. (D) Western blot analyses of the levels of FRQ protein in the wild-type and *cbf-1*^*KO*^ strains. Asterisks indicate nonspecific bands. Samples were grown in DD for indicated hours before harvest. PVDF membrane stained with Coomassie blue (mem) was used as a loading control. Quantification of the levels of FRQ protein is shown beside the western blot. (E) Northern blot analyses of the levels of *frq* mRNA. Ribosome RNA (rRNA) bands stained by ethidium bromide shown below the northern blot acted as a loading control for each sample. Quantification of the levels of *frq* mRNA is shown beside the northern blot.

To identify new components that regulate the transcription of clock gene *frq* in *N*. *crassa*, we generated viable knockout mutants of transcription factors and performed race tube assays to screen for mutants with defects in circadian conidiation rhythms. We found that the deletion of *cbf-1* gene (NCU08999) resulted in 2-hour longer period and much lower amplitude of circadian conidiation rhythm than those of the wild-type strain (Fig 1A). To confirm the period of the *cbf-1* mutant at the molecular level, we introduced a plasmid that carries a luciferase reporter construct (*frq-luc*) into the *cbf-1*^*KO*^ strain at the *his-3* locus. As shown in Fig 1B, the bioluminescence rhythm of the *cbf-1*^*KO*^, *frq-luc* strains was of very low amplitude and long period comparing to the *wt*, *frq-luc* strains. Sequence alignment revealed that the helix-loop-helix (HLH) region of CBF-1 protein is highly conserved from yeast to mammals (Fig 1C).

To determine how CBF-1 influences the circadian clock, we examined the FRQ expression profile at different time points in constant darkness (DD). FRQ protein levels were lower in the *cbf-1*^*KO*^ strain than in the wild-type strain in DD. Moreover, the peak of FRQ protein was delayed in the *cbf-1*^*KO*^ strain relative to the wild-type strain (Fig 1D), which is consistent with its long period phenotype. The levels of *frq* mRNA in the *cbf-1*^*KO*^ strain were increased in DD (Fig 1E), suggesting that CBF-1 suppresses *frq* transcription. We then evaluated FRQ stability after the addition of the protein synthesis inhibitor cycloheximide (CHX). FRQ stability was similar in the *cbf-1*^*KO*^ strain and the wild-type strain (S1 Fig). The mechanism that results in low FRQ protein levels but high *frq* mRNA levels in the *cbf-1*^*KO*^ strain is unknown, but these molecular data indicate that the circadian clock is dampened in the *cbf-1*^*KO*^ strain due to impaired regulation of *frq* expression.

### Overexpression of CBF-1 leads to low amplitude rhythms

**Fig 2:**
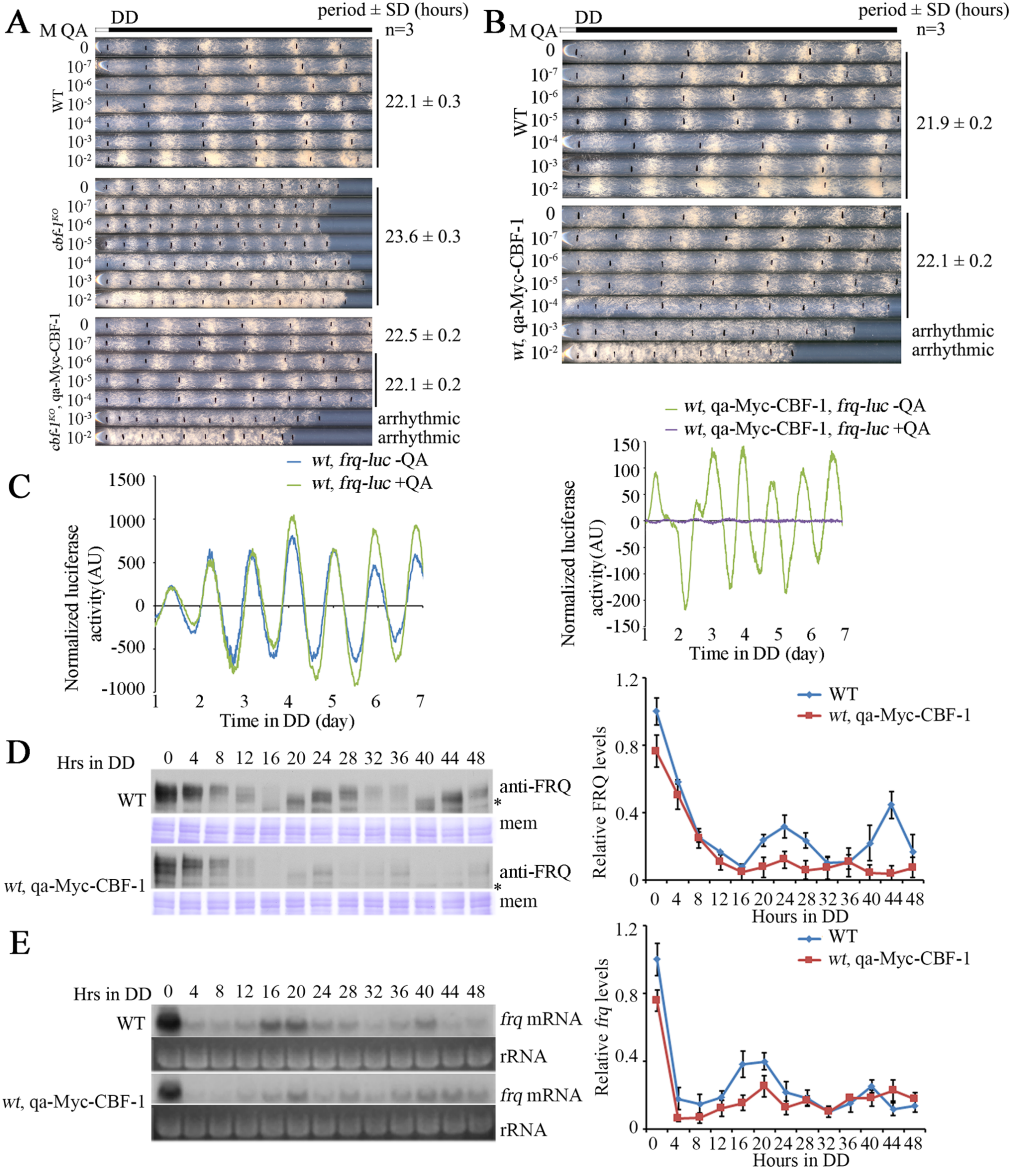
Overexpression of CBF-1 in the wild-type strain abolishes conidiation rhythmicity. (A) The leaky expression of CBF-1 can partially rescue the *cbf-1*^*KO*^ phenotype. The highly inducible Myc-CBF-1 caused an arrhythmic conidiation phenotype. (B) The QA-regulated phenotype of wild-type and *wt*, qa-Myc-CBF-1 strains is shown on race tubes containing variable amounts of the inducer QA (0-10^-2^ M). (C) Luciferase reporter assays showing the normalized *frq* promoter activity of *wt*, *frq*-*luc* and *wt*, qa-Myc-CBF-1, *frq*-*luc* strains. (D) Western blot analyses of the levels of FRQ protein in the wild-type and *wt*, qa-Myc-CBF-1 strains. Asterisks indicate nonspecific bands. PVDF membranes stained with Coomassie blue (mem) demonstrate equal amounts of protein loaded per lane. Quantification of the levels of FRQ protein is shown beside the western blot. (E) Northern blot analyses of the levels of *frq* mRNA in the wild-type and *wt*, qa-Myc-CBF-1 strains. rRNA bands stained with ethidium bromide shown below the northern blot served as a loading control for each sample. Quantification of the levels of *frq* mRNA is shown beside the northern blot.

To further confirm the function of CBF-1 in the circadian clock, a construct in which the expression of Myc-tagged CBF-1 is driven by quinic acid (QA)-inducible *qa-2* promoter was introduced to the *cbf-1*^*KO*^ strain. We quantified Myc-CBF-1 expression in growth medium containing different QA concentrations (0 to 10^−2^ M). The amount of Myc-CBF-1 with 10^−7^ M QA and without QA (S2A Fig) partially restore the conidiation rhythm of *cbf-1* mutant (Fig 2A), indicating that the low levels of Myc-CBF-1 partially rescue the circadian conidiation defects of *cbf-1*^*KO*^ strain. The conidiation rhythm of the *cbf-1*^*KO*^, qa-Myc-CBF-1 strain was completed rescued when the QA concentration was between 10^−6^ and 10^−4^ M in the medium. QA at 10^−3^ M, however, resulted in a slow growth rate and arrhythmic conidiation rhythm in the *cbf-1*^*KO*^, qa-Myc-CBF-1 strain (Fig 2A), suggesting that the proper level of CBF-1 is critical for circadian clock function.

QA-inducible gene expression was known to be suppressed by catabolites [38]. To determine whether the leaky expression of Myc-CBF-1 is subjected to catabolite repression, we evaluated the conidiation rhythm of the *cbf-1*^*KO*^, qa-Myc-CBF-1 strain in different concentrations of glucose in the race tube medium without QA. The race tube phenotype of the *cbf-1*^*KO*^, qa-Myc-CBF-1 strain was not affected by glucose concentration (S3 Fig), suggesting that the leaky expression of Myc-CBF-1 in the absence of QA is probably unrelated to a glucose effect.

To verify whether the levels of CBF-1 protein are important for conidiation rhythm, the pqa-Myc-CBF-1 plasmid was introduced into the wild-type stain. Addition of QA (0 to 10^−2^ M) resulted in increased levels of Myc-CBF-1 in a dose-dependent manner (S2B Fig). As expected, the race tube assay showed that QA (10^−3^ M) induced expression of high levels of Myc-CBF-1 in the *wt*, qa-Myc-CBF-1 strain and that the overexpression of Myc-CBF-1 resulted in growth and circadian conidiation defects (Fig 2B). To further confirm these results, we introduced a luciferase reporter construct (*frq-luc*) into the *wt*, qa-Myc-CBF-1 strain. Overexpression of CBF-1 in the wild-type strain resulted in very low amplitude circadian bioluminescence rhythm (Fig 2C). Thus, CBF-1 is critical for the clock function and high levels of CBF-1 protein interfere with growth and circadian clock function in *N*. *crassa*.

We then examined the rhythmic expression of FRQ protein and *frq* mRNA in the wild-type and *wt*, qa-Myc-CBF-1 strains. High levels of Myc-CBF-1 were induced in the presence of 10^−3^ M QA (S2C Fig) at different time points in DD. Overexpression of Myc-CBF-1 caused a marked decrease in FRQ expression, and FRQ cycling amplitude was also affected (Fig 2D). Northern blot showed that the level of *frq* mRNA was also reduced in the *wt*, qa-Myc-CBF-1 strain compared to that in wild-type strain in the first 24 hours (Fig 2E), indicating that CBF-1 suppresses *frq* transcription. After 24 hours in DD, however, the level of *frq* mRNA was comparable in mutant and wild-type strains, suggesting that overexpressed CBF-1 also affected FRQ expression at a post-transcriptional level.

The low levels of FRQ protein in the *wt*, qa-Myc-CBF-1 strain promoted us to examine the expression of WC-1 and WC-2 in this strain. The levels of WC-1 and WC-2 proteins in the *wt*, qa-Myc-CBF-1 strain were lower than those in the wild-type strain (S4A Fig). However, the levels of *wc-1* and *wc-2* mRNA were similar (S4B Fig). These results suggest that overexpressed CBF-1 negatively regulates the levels of WC-1 and WC-2 proteins in a post-transcriptional manner.

### Rhythmic CBF-1 association with the C-box of the *frq* promoter is regulated by WCC activity

**Fig 3:**
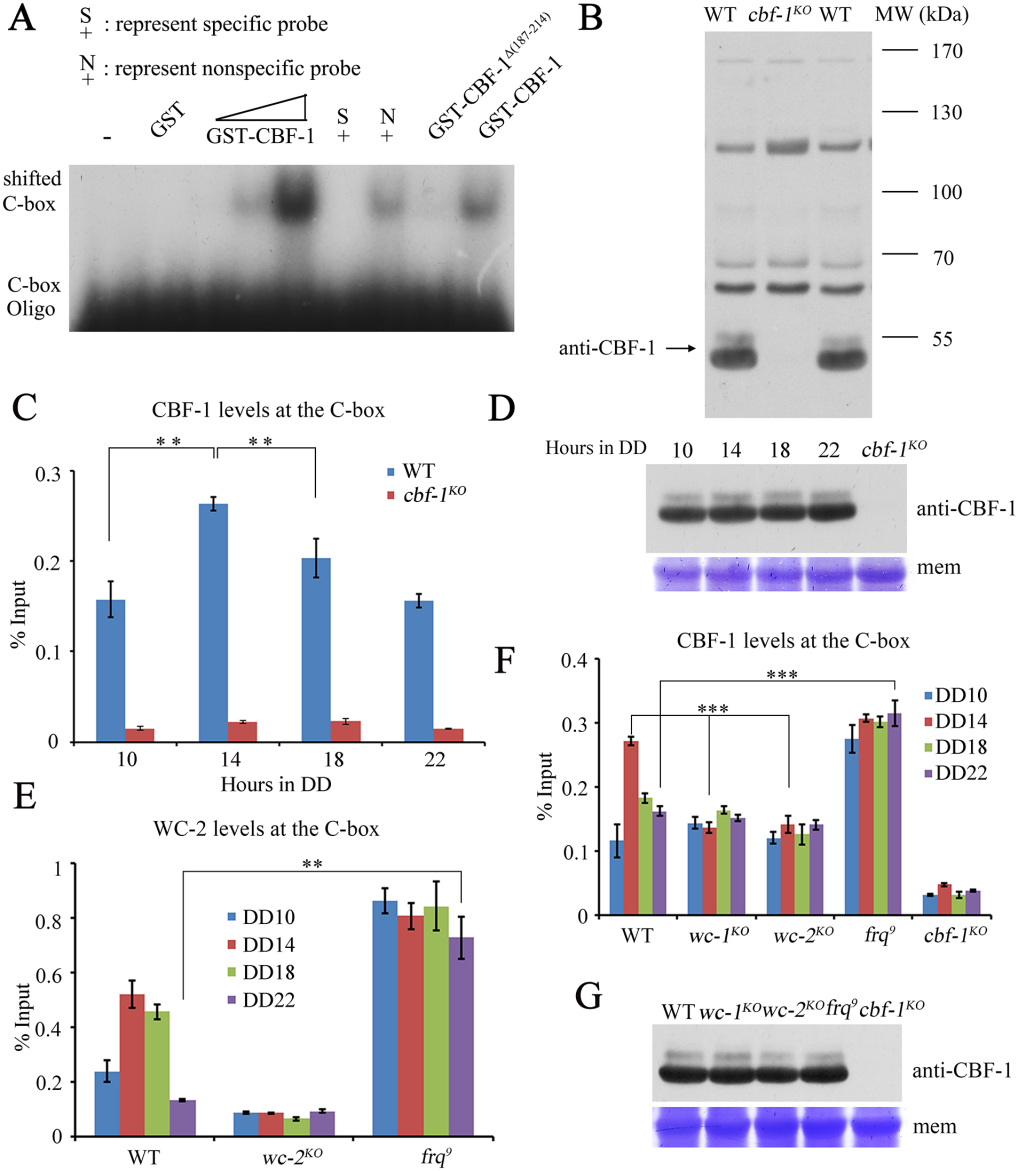
CBF-1 rhythmically associates with the C-box of the *frq* promoter. (A) EMSA assays showing GST-CBF-1 specifically binds to the C-box. A lower concentration of protein was utilized when protein was incubated with specific and non-specific probe. (B) Western blot analyses showing that polyclonal antibody specifically recognizes CBF-1 protein in the wild-type strain but not in the *cbf-1*^*KO*^ strain. The arrow indicates the CBF-1 protein band detected by CBF-1 polyclonal antibody. WT, wild-type; MW, molecular weight. (C) CBF-1 rhythmically binds to the C-box of *frq* promoter. Samples were grown for the indicated hours in DD prior to harvest for ChIP. (D) The levels of CBF-1 protein are consistent across circadian time points. (E) Rhythmic binding of WC-2 to the C-box is constantly high in the *frq*^*9*^ mutant. (F) Rhythmic binding of CBF-1 to the C-box is disrupted in *wc-1*, *wc-2*, and *frq*^*9*^ mutants. (G) The levels of CBF-1 protein were not altered in *wc-1*, *wc-2*, or *frq*^*9*^ mutants. Significance was assessed by using a two-tailed *t*-test. Data are means ± standard deviations (S.D.) (n = 3; **P<0.01 and ***P<0.001).

To further investigate whether CBF-1 directly regulates *frq* transcription by binding to the *frq* promoter, we performed electrophoretic mobility shift (EMSA) and chromatin immunoprecipitation (ChIP) assays. For the EMSA assay, purified GST-CBF-1 or GST-CBF-1^Δ(187–214)^ fusion proteins or GST only (S5A Fig) were incubated with a radioactively labeled C-box oligonucleotide probe. A migrating complex was observed when the GST-CBF-1 fusion protein was present, but not when GST or the GST-CBF-1^Δ(187–214)^ fusion proteins were used (Fig 3A). The interaction was specific for the C-box sequence as an unlabeled C-box oligonucleotide disrupted the complex, but an oligonucleotide with a different sequence did not (Fig 3A). These results suggest that CBF-1 binds directly to the C-box of *frq* promoter sequence.

To test whether CBF-1 binds to the *frq* promoter *in vivo*, we generated a CBF-1-specific antibody, which recognized a specific band at predicted molecular weight in the wild-type strain but not in the *cbf-1*^*KO*^ strain (Fig 3B). A ChIP assay using this antibody showed that the enrichment of CBF-1 at the C-box of *frq* promoter was specific and rhythmic, peaking at DD14 when *frq* transcription and WCC binding are high (Fig 3C). The levels of CBF-1 protein, however, were constant (Fig 3D).

Because both CBF-1 and WCC are transcription factors and rhythmically bind to the C-box, we evaluated the relationship between the two factors in *frq* promoter binding. We examined the binding of CBF-1 to the *frq* promoter in *wc-1*^*KO*^, *wc-2*^*KO*^, and *frq*^*9*^ mutant strains. In the latter, a frame-shift mutation of *frq* results in truncated FRQ protein and defective negative feedback loop [39]. The binding of CBF-1 to the C-box was constantly low in the *wc-1*^*KO*^ and *wc-2*^*KO*^ strains but was constantly high in the *frq*^*9*^ mutant strain with high levels WCC activity (Fig 3E and 3F). Moreover, the CBF-1 protein levels were not altered in these mutants (Fig 3G). These data suggested that the rhythmic CBF-1 association with the *frq* promoter is regulated by WCC activity.

### CBF-1 suppresses WC-independent *frq* transcription by promoting the recruitment of the transcriptional co-repressor RCM-1

**Fig 4:**
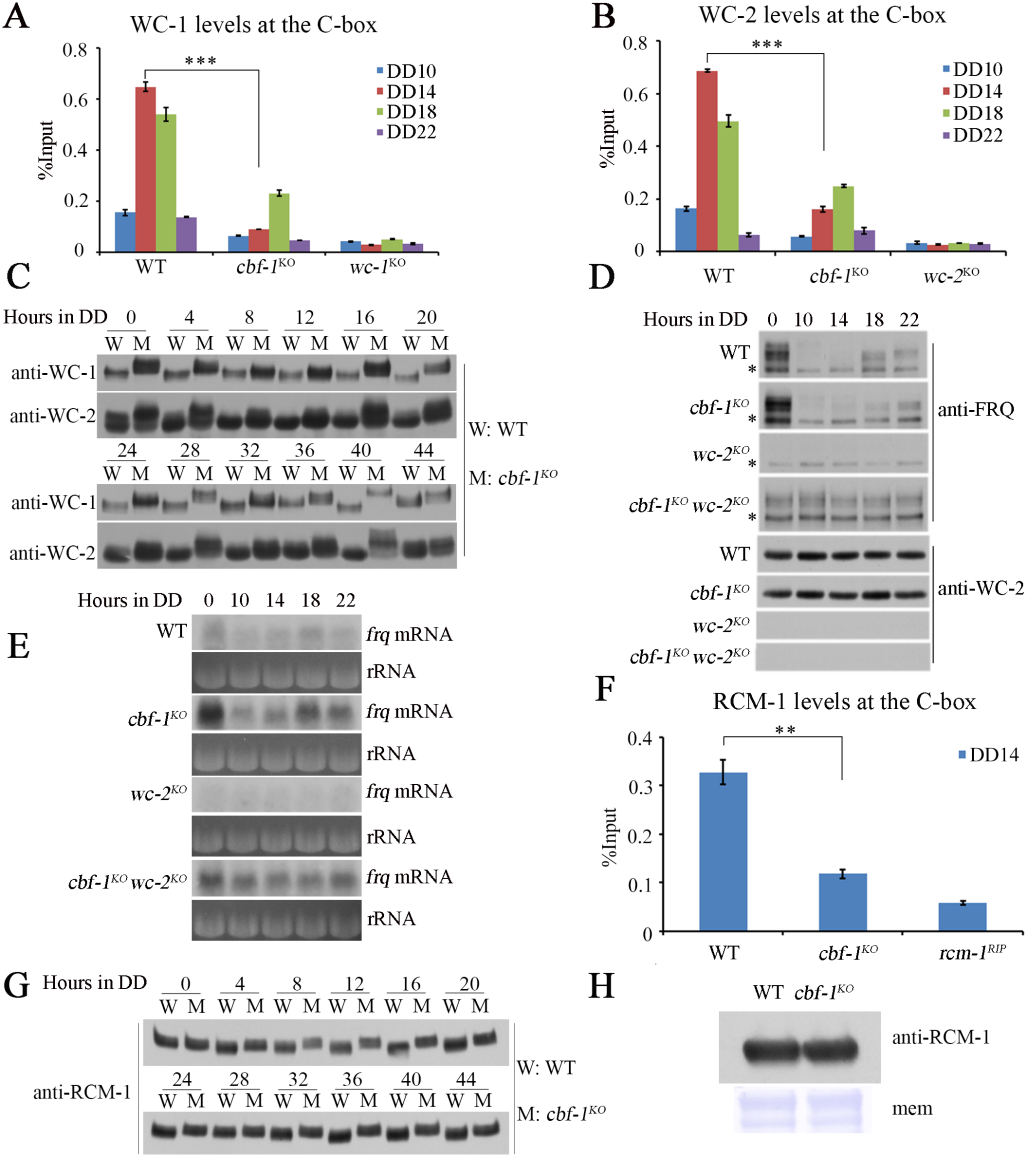
CBF-1 suppresses WC-independent *frq* transcription by promoting the recruitment of the transcriptional co-repressor RCM-1. (A, B) ChIP assays showing the enrichment of A) WC-1 and B) WC-2 is decreased and the peak of WC-1 binding is delayed in the *cbf-1*^*KO*^ strain compared to the wild-type strain. (C) The phosphorylation of WC-1 and WC-2 was markedly increased over time in the *cbf-1*^*KO*^ strain compared to the wild-type strain. (D) Western blot analyses were performed using antibodies against FRQ or WC-2 in the wild-type, *cbf-1*^*KO*^, *wc-2*^*KO*^, and *cbf-1*^*KO*^ *wc-2*^*KO*^ strains. Samples were grown under constant light (LL) or in DD for indicated hours before harvest. (E) Northern blot analyses of the levels of *frq* mRNA in wild-type, *cbf-1*^*KO*^, *wc-2*^*KO*^, and *cbf-1*^*KO*^ *wc-2*^*KO*^ strains. (F) The enrichment of RCM-1 at the C-box of *frq* promoter is reduced in *cbf-1*^*KO*^ strains at the DD14 compared to the wild-type strain. (G) The levels of RCM-1 phosphorylation of the *cbf-1*^*KO*^ strain are higher than those in wild-type strain at the indicated times. (H) The RCM-1 protein levels are similar to that in the *cbf-1*^*KO*^ and wild-type strains. The errors bars ±S.D. (n = 3; **P<0.01 and ***P<0.001; two-tailed *t*-test).

To determine how CBF-1 regulates *frq* transcription, we performed ChIP assays using WC-1 and WC-2 antibodies [27]. Our results showed that WCC rhythmically bound to the C-box of the *frq* promoter in DD with a peak at DD14 in wild-type strain (Fig 4A and 4B). However, the robust rhythmic binding of WCC to the C-box was dramatically decreased with a low amplitude and delayed peak in the *cbf-1*^*KO*^ strain (Fig 4A and 4B). Previous studies showed that hypophosphorylated WC-1 and WC-2 efficiently bound to the C-box activating *frq* transcription [16, 17] and that hyperphosphorylated WC-1 and WC-2 had lower affinity for the C-box of *frq* promoter in DD [40]. Western blots showed that WC-1 and WC-2 were hyperphosphorylated in the *cbf-1*^*KO*^ strain compared with the wild-type strain throughout DD (Fig 4C). However, we observed no significant differences in levels of WC-1 or WC-2 in the *cbf-1*^*KO*^ strain compared to those in the wild-type strain (S5B Fig). These results suggest that the loss of CBF-1 decreases WCC activity by promoting phosphorylation of WC-1 and WC-2. A previous study showed that hyperphosphorylation of WC-1 and WC-2, which was mediated by FRQ, resulted in less binding to the C-box of *frq* [41]. Thus, the low WCC activity in the *cbf-1*^*KO*^ strain may be mediated by FRQ.

The decreased binding of WC-1 and WC-2 at C-box of *frq* promoter and the high levels of *frq* mRNA in the *cbf-1*^*KO*^ strain prompted us to examine whether there is WC-independent *frq* transcription in the *cbf-1*^*KO*^ mutant. Thus, we generated the *cbf-1*^*KO*^ *wc-2*^*KO*^ double mutant and compared FRQ levels with those in the *wc-2*^*KO*^ single mutant. As expected, constant levels of FRQ expression were observed in *cbf-1*^*KO*^ *wc-2*^*KO*^ double mutant but not in *wc-2*^*KO*^ single mutant (Fig 4D). Further, constant high levels of *frq* mRNA were also observed in the *cbf-1*^*KO*^ *wc-2*^*KO*^ double mutant (Fig 4E), indicating that CBF-1 is required for suppression of WC-independent transcription of *frq*. These results indicate that the constant expression of FRQ in the *cbf-1*^*KO*^ strain mediates the hyperphosphorylation of WC proteins, resulting in decreased binding of WCC to the C-box of the *frq* promoter. WC-independent *frq* expression leads to the increased *frq* mRNA level in the *cbf-1* mutant. However, low *luc* expression levels in the *cbf-1*^*KO*^, *frq-luc* strains suggest the lack of WC-independent transcription. The entire ORF and 3’UTR of *frq* are replaced by *luc* in *frq-luc* transgene and the *frq-luc* transgene is targeted to the *his-3* locus. So, the lack of WC-independent transcription of the *frq-luc* reporter may be caused by the chromatin state of the *frq*-luc locus being different from the chromatin state of the *frq* locus [27].

Our previous study showed that the transcriptional co-repressor RCM-1 suppresses WC-independent *frq* transcription by binding to the *frq* locus and that hyperphosphorylation of RCM-1 impairs its suppressor activity [28]. The level of RCM-1 enrichment at the C-box of *frq* promoter was dramatically reduced and RCM-1 became hyperphosphorylated in the *cbf-1*^*KO*^ strain compared to the wild-type strain (Fig 4F and 4G), but the levels of RCM-1 were similar in the two strains (Fig 4H). Therefore, the WC-independent *frq* transcription in the *cbf-1*^*KO*^ strain may be caused at least partially by hyperphosphorylation of RCM-1.

### CBF-1 suppresses WCC binding in the absence of FRQ feedback inhibition

**Fig 5:**
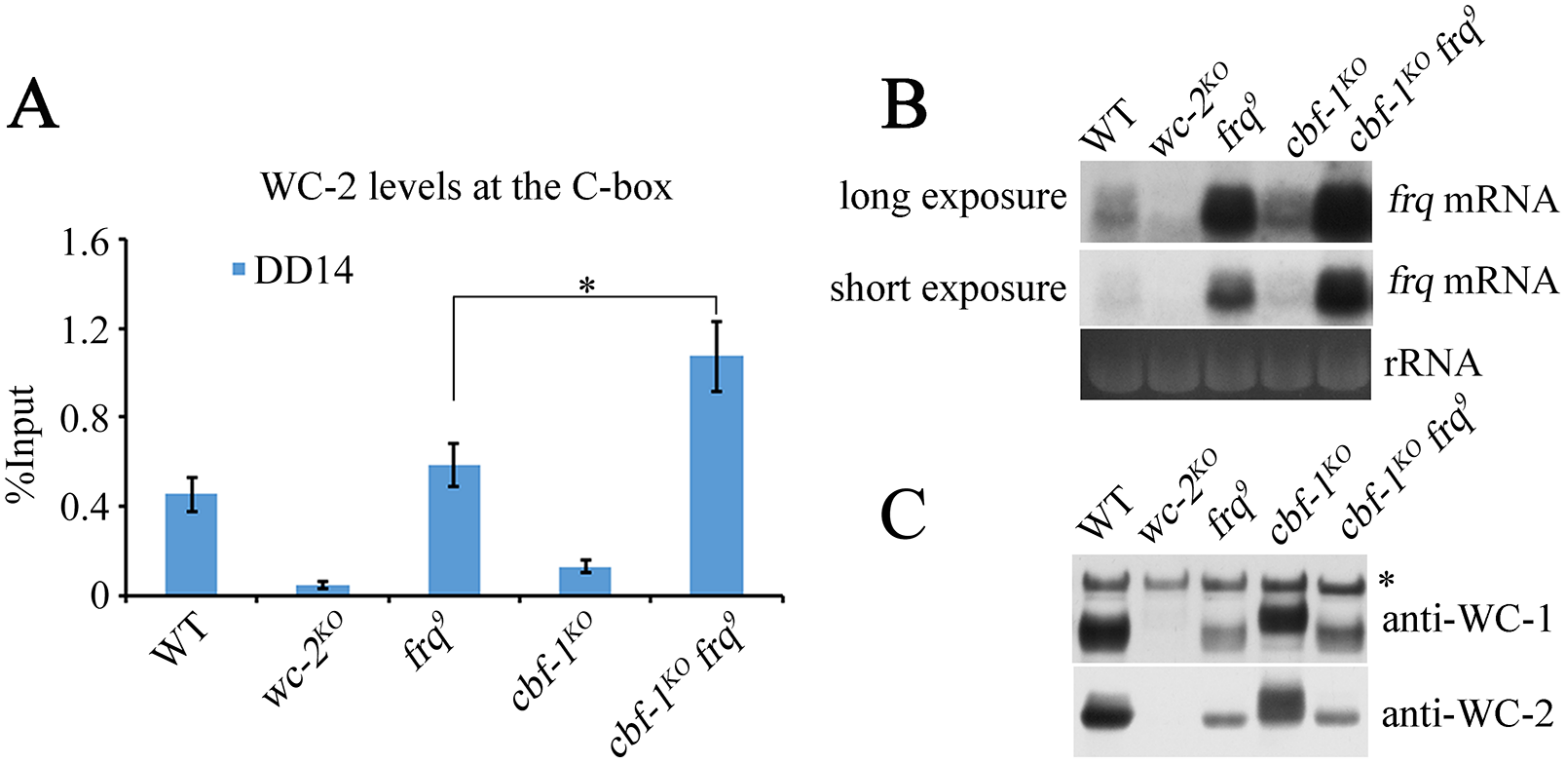
CBF-1 suppresses WCC binding without FRQ-feedback inhibition. (A) The recruitment of WC-2 to the C-box of the *frq* promoter in the wild-type, *wc-2*^*KO*^, *frq*^*9*^, *cbf-1*^*KO*^, and *cbf-1*^*KO*^ *frq*^*9*^ strains at DD14. (B) Northern blot analysis of the levels of *frq* mRNA in wild-type, *wc-2*^*KO*^, *frq*^*9*^, *cbf-1*^*KO*^ and *cbf-1*^*KO*^ *frq*^*9*^ strains. (C) The phosphorylation levels of WC-1 and WC-2 do not change between *frq*^*9*^ and *cbf-1*^*KO*^ *frq*^*9*^ strains. Plotted are means ±S.D. (n = 3; **P<0.01 and ***P<0.001; two-tailed *t*-test).

Our results suggest that the decreased binding of WCC to the C-box in the *cbf-1*^*KO*^ strains is due to FRQ-mediated hyperphosphorylation of WCC. To further test whether CBF-1 directly regulates WCC binding of the C-box independent of FRQ, we generated the *cbf-1*^*KO*^ *frq*^*9*^ double mutant (S6A Fig). A ChIP assay showed that WC-2 was significantly enriched at the C-box of the *cbf-1*^*KO*^ *frq*^*9*^ double mutant compared to the *frq*^*9*^ single mutant (Fig 5A) even though the WC levels were similar in the two strains (S6B Fig). The results indicate that CBF-1 can suppress WC-2 binding at the C-box independently of FRQ. The levels of *frq* mRNA were higher in the *cbf-1*^*KO*^ *frq*^*9*^ double mutant than in the *frq*^*9*^ single mutant (Fig 5B). WC-1 and WC-2 were both hypophosphorylated in the *cbf-1*^*KO*^ *frq*^*9*^ double mutant and in the *frq*^*9*^ single mutant (Fig 5C), suggesting that the increased binding of WC-2 to the *frq* C-box was due to the absence of CBF-1 protein but was not affected by WC phosphorylation status. Taken together, these results suggest that CBF-1 suppresses WCC binding to the C-box of *frq* promoter through a FRQ-independent mechanism.

### The HLH domain of CBF-1 is required for binding to the C-box of *frq* promoter

**Fig 6:**
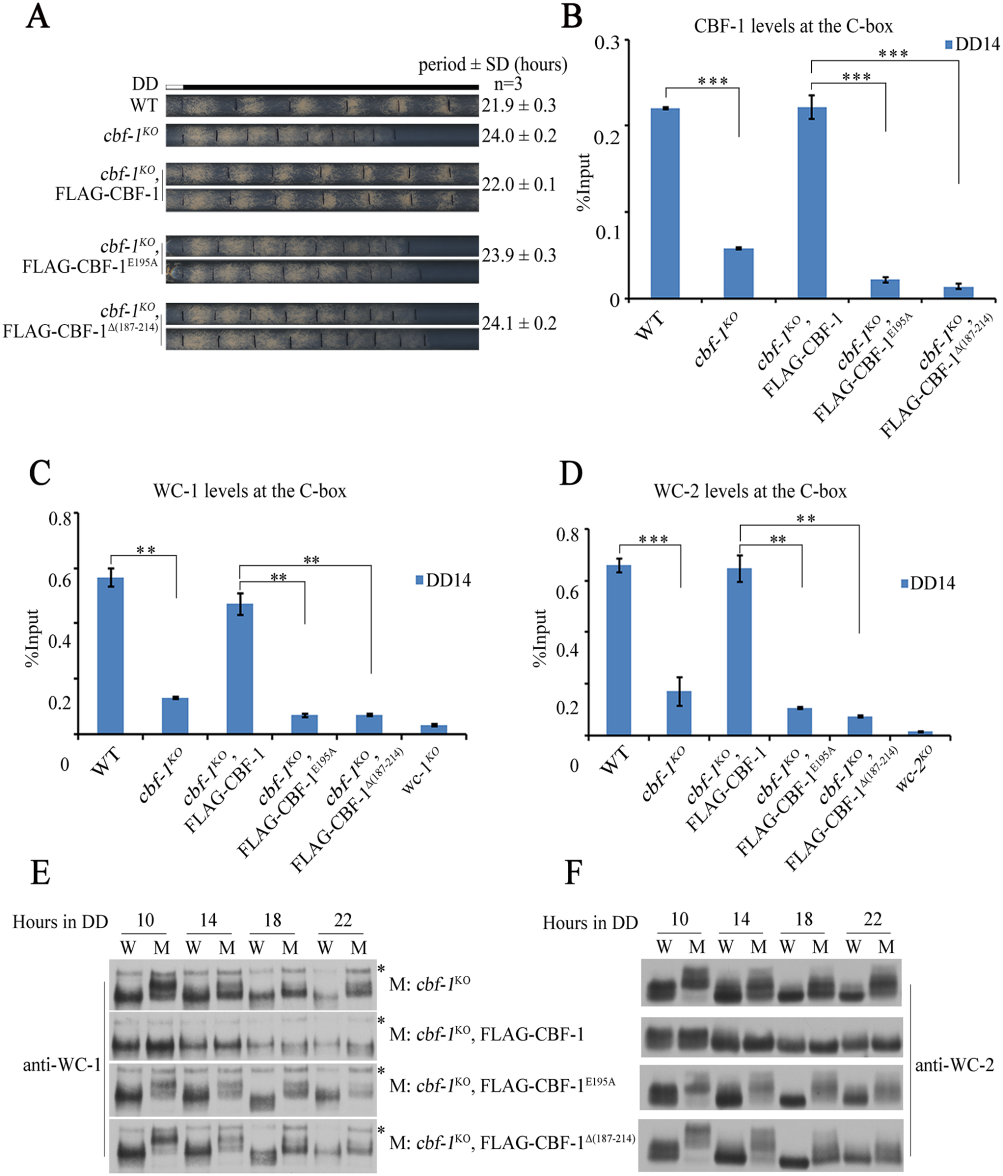
Conserved HLH domain of CBF-1 is required for normal circadian gene expression. (A) Race tube assays showing that the defect of circadian conidiation rhythms in *cbf-1* mutants can be rescued by wild-type CBF-1 but not mutant CBF-1 proteins. (B) The enrichment of CBF-1 at the C-box is abolished in mutants of CBF-1 defective in DNA binding. (C, D) ChIP assays of C) WC-1 and D) WC-2 were performed in the indicated strains at DD14. (E, F) The phosphorylation levels of E) WC-1 and F) WC-2 were markedly increased in mutants of CBF-1 defective in DNA binding. Plotted are means ±S.D. (n = 3; **P<0.01 and ***P<0.001; two-tailed *t*-test).

The HLH DNA binding domains are conserved in all CBF-1 homologues from yeast to human (Fig 1C). To test the role of CBF-1 binding to the *frq* promoter, we generated FLAG-tagged CBF-1 constructs that contain a E195A point mutation or deletion of the 187–214 amino acid region in the HLH domain [42]. Unlike the wild-type FLAG-CBF-1 protein, the mutant FLAG-CBF-1 proteins failed to rescue the long period conidiation phenotype of the *cbf-1*^*KO*^ strain (Fig 6A and S7A Fig), suggesting that the DNA binding activity of CBF-1 is required for its circadian clock function. A ChIP assay showed that CBF-1 enrichment at the C-box in *cbf-1*^*KO*^, p*cbf-1*-FLAG-CBF-1^E195A^, and *cbf-1*^*KO*^, p*cbf-1*-FLAG-CBF-1^Δ(187–214)^ strains was abolished (Fig 6B). These results demonstrate that CBF-1 regulates the circadian clock via its HLH DNA binding domain. Similarly, neither the defect of WCC binding to the C-box nor the hyperphosphorylation of WC-1 and WC-2 was rescued by mutant FLAG-CBF-1 proteins (Fig 6C–6F). The levels of WC proteins were not affected in the *cbf-1*^*KO*^ mutants (S7B and S7C Fig). These results suggest that CBF-1 binding at the C-box of *frq* promoter is required for its role in the circadian clock.

### Overexpression of CBF-1 decreases WCC recruitment to the C-box of the *frq* promoter

**Fig 7:**
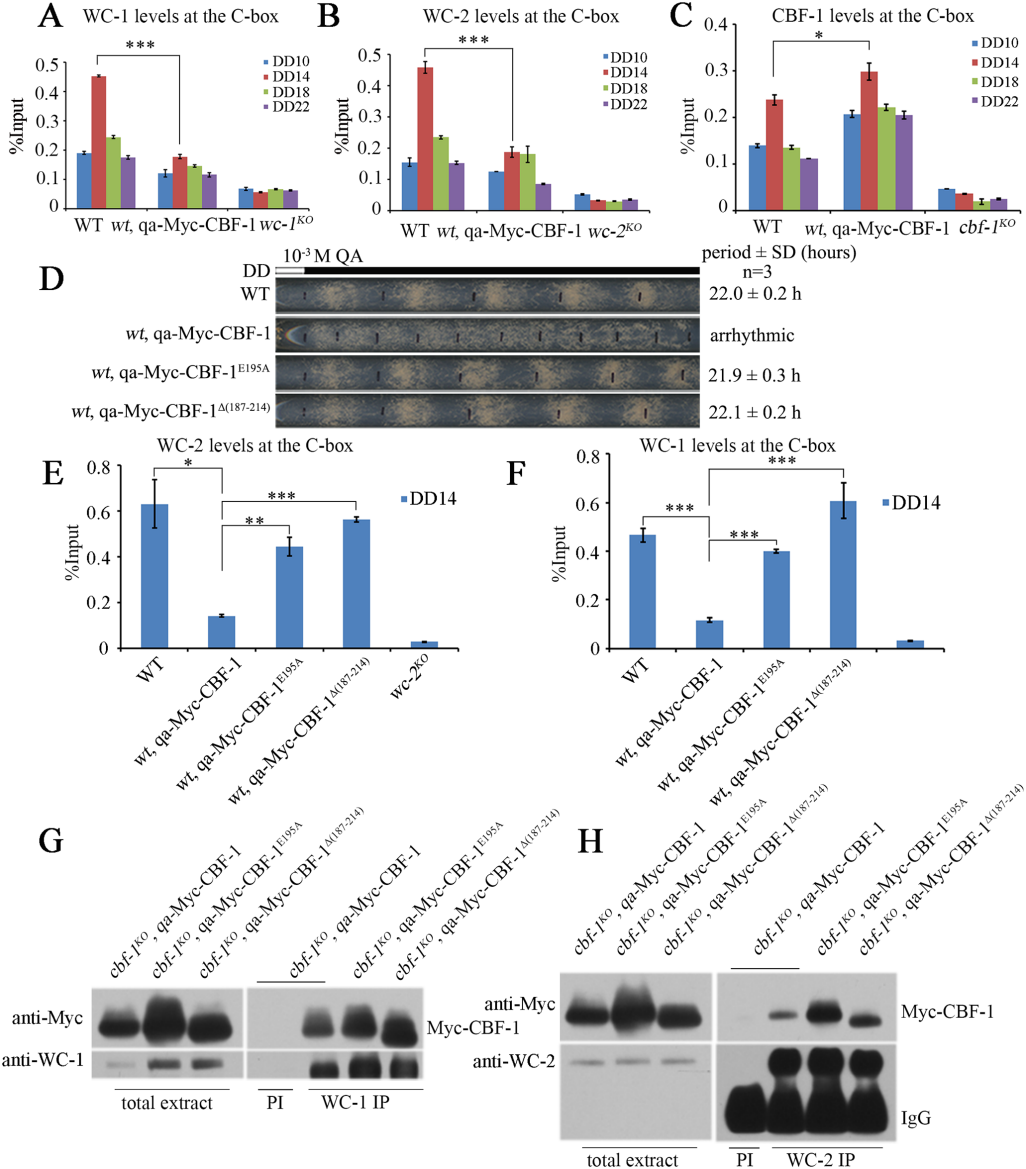
Overexpression of CBF-1 decreases recruitment of WCC to the C-box. (A, B) Rhythmic binding of A) WC-1 and B) WC-2 to the C-box is decreased by overexpression of Myc-CBF-1. (C) ChIP assays showing that the binding of CBF-1 to C-box increased in wild-type strain with overexpression of CBF-1. (D) Race tube assays showing that overexpression of the mutant CBF-1 has no effect on circadian conidiation phenotype. (E, F) ChIP assays showing that the binding of E) WC-1 and F) WC-2 decreased in the wild-type strain upon overexpression of the wild-type CBF-1 but not mutant CBF-1 at DD14. All error bars represent the ±S.D. (n = 3; *P<0.05, **P<0.01, and ***P<0.001; two-tailed *t*-test). (G, H) Immunoprecipitation assays using G) WC-1 antiserum and H) WC-2 antiserum showing that different versions of CBF-1 proteins interact with WC-1 and WC-2 *in vivo*.

To determine whether the decreased levels of *frq* mRNA in the *wt*, qa-Myc-CBF-1 strain is caused by impaired WCC recruitment to the C-box of *frq* promoter, we performed ChIP assays with WC-1 and WC-2 antibodies. ChIP data showed that robust rhythmic binding of WCC to the C-box was markedly decreased by the overexpression of Myc-CBF-1 in the *wt*, qa-Myc-CBF-1 strain compared to the wild-type strain (Fig 7A and 7B). In contrast, ChIP assays with CBF-1 antibody showed that the recruitment of CBF-1 to the C-box of *frq* promoter was increased in the *wt*, qa-Myc-CBF-1 strain (Fig 7C). These results suggest that overexpression of CBF-1 interferes with WC recruitment to the C-box.

To confirm this conclusion, we created a *wt*, qa-Myc-CBF-1^E195A^ strain and a *wt*, qa-Myc-CBF-1^Δ187–214^ strain. As expected, the circadian conidiation phenotype was not affected by overexpression of these mutant Myc-CBF-1 proteins that cannot bind to *frq* C-box (Fig 7D). Furthermore, ChIP assays with WC-1 and WC-2 antibodies showed that binding of WCC to the C-box was similar in the *wt*, qa-Myc-CBF-1^E195A^ and *wt*, qa-Myc-CBF-1^Δ(187–214)^ strains to that in the wild-type strain (Fig 7E and 7F). In addition, the levels of WCC and FRQ proteins were not affected in the *wt*, qa-Myc-CBF-1^E195A^ or *wt*, qa-Myc-CBF-1^Δ187–214^ strains (S8 Fig). Together, these results are consistent with a model in which CBF-1 binding to the C-box region impairs WCC binding.

As WCC and CBF-1 appear to be mutual regulators in *frq* promoter binding, we tested whether they interact. Co-immunoprecipitation assays were performed using pre-immune serum as the negative control. We found that CBF-1 co-immunoprecipitated with WC-1 and WC-2, suggesting that these proteins interact to regulate *frq* transcription (Fig 7G and 7H). Taken together, our results suggest that the proper level of CBF-1 is critical for modulating the binding of WCC at the C-box of the *frq* promoter to allow rhythmic WC-dependent *frq* transcription.

## Discussion

Transcriptional control of circadian clock genes is an essential step in negative feedback loops of all eukaryotic clock systems. Previous studies have demonstrated PAS domain-containing transcription factors, such as WC-1 and WC-2 in *N*. *crassa* and CLOCK and BMAL1 in mammals, are responsible for rhythmically activating clock gene transcription [43–45]. In this study, we found that CBF-1, a helix-loop-helix domain-containing transcription factor, is also involved in regulating *frq* transcription in *N*. *crassa*. In the *cbf-1*^*KO*^ strain, the circadian conidiation rhythm was severely affected, and the FRQ protein oscillation was delayed in DD. Overexpression of CBF-1 resulted in low amplitude rhythms, decreased levels of *frq* mRNA, and reduced WCC binding to the C-box of the *frq* promoter. Finally, rhythmic binding of WCC to the C-box of *frq* promoter required functional CBF-1. Taken together, our results suggest that CBF-1 is critical for robust rhythmic *frq* transcription.

**Fig 8:**
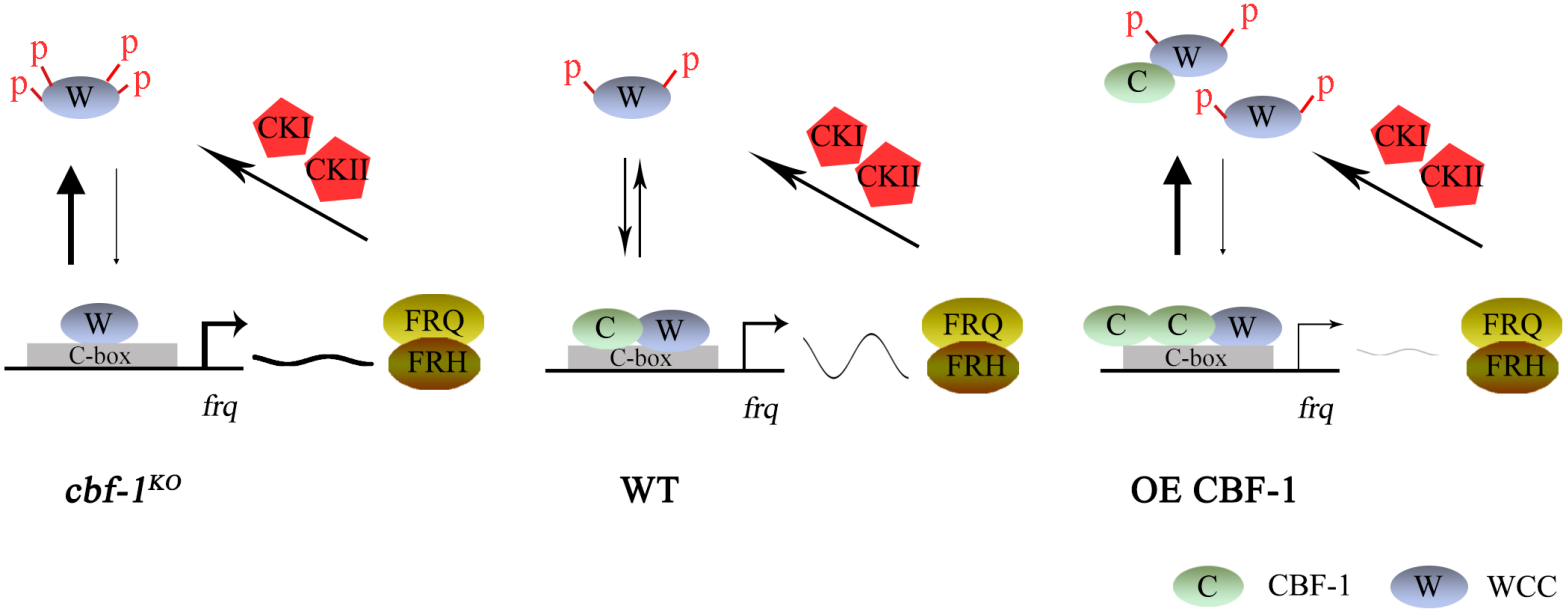
A model of CBF-1-mediated regulation of *frq* transcription. In the wild-type strain, WCC and CBF-1 bind to the C-box site in the regulatory region of the *frq* gene and cooperate with each other to ensure rhythmic expression of FRQ. In the *cbf-1*^*KO*^ mutant, the delay in WCC binding to the C-box may lead to long periods without CBF-1. WC-dependent and WC-independent *frq* transcription both contribute to the levels of *frq* mRNA. In the CBF-1 overexpression strain, elevated CBF-1 binding to the C-box decreases the recruitment of WCC, which leads to *frq* mRNA oscillation at a low level.

The rhythmic association of CBF-1 with the C-box in the *frq* promoter is regulated by WCC activity. Rhythmic binding of CBF-1 to the C-box was disrupted in *wc-1*, *wc-2*, and *frq*^*9*^ mutants (Fig 3F), but high levels of WCC activity and high CBF-1 recruitment were observed in the *frq*^*9*^ strain (Fig 3E and 3F). Our data suggest that CBF-1 has dual functions in the circadian clock both by influencing on WCC binding at the *frq* promoter and by suppressing WC-independent FRQ expression. In the wild-type strain, both WCC and CBF-1 rhythmically bind to the C-box of *frq* promoter to activate *frq* transcription (Fig 8). FRQ protein then promotes phosphorylation of the WCC by CKI and CKII kinases, leading to its inactivation and inhibition of *frq* transcription. FRQ is progressively phosphorylated by CKI and CKII kinases and degraded. After FRQ degrades to a certain level, WCC is reactivated and the cycle restarts. In the *cbf-1*^*KO*^ strains, WC-independent *frq* transcription is activated, which promotes WCC phosphorylation and inhibits WCC binding to the C-box, resulting in low amplitude and long period phenotype. As shown in Fig 4D and 4E, constant intermediate levels of *frq* mRNA and FRQ protein were detected in the *wc-2*^*KO*^ *cbf-1*^*KO*^ double mutant, indicating the activation of WC-independent *frq* transcription in *cbf-1*^*KO*^ mutants. Consistent with this notion, constant high levels of *frq* mRNA were observed in DD in the *cbf-1*^*KO*^ mutant (Fig 1E). In the CBF-1 overexpression strain, we observed elevated CBF-1 binding to C-box region but reduced WCC recruitment, resulting in low level of *frq* mRNA and low amplitude rhythm. Because both CBF-1 and WCC bind to C-box in the *frq* promoter and CBF-1 can also bind to C-box independent of WCC, it is possible that high CBF-1 level inhibits WCC C-box binding through competitive binding to C-box. In addition, the reduced WC levels in the CBF-1 overexpression strain can also contribute to the reduced WCC binding to C-box (S4A Fig). Therefore, CBF-1 protein levels must be tightly regulated to allow robust rhythmic WC-dependent *frq* transcription.

A role for CBF-1 in regulating *frq* transcription by modulating WCC binding at the *frq* promoter is supported by several lines of evidence. First, EMSA and ChIP assays showed that CBF-1 rhythmically binds to the C-box region *in vitro* and *in vivo* (Fig 3A and 3C). Second, the binding of WCC was significantly higher in the *frq*^*9*^ *cbf-1*^*KO*^ double mutant than that in *frq*^*9*^ single mutant, suggesting that CBF-1 can suppress WCC binding to the C-box independently of FRQ (Fig 5A). Third, even though CBF-1 and WCC interacts and WCC promotes the C-box binding of CBF-1, CBF-1 can also bind to C-box independent of WCC (Fig 3A). As a result, overexpression of CBF-1 led to increased CBF-1 binding to the C-box region but decreased WCC binding (Fig 7C, 7E and 7F). Together, these results suggest that CBF-1 binding to the C-box region impairs WCC binding to the C-box region of the *frq* promoter.

Our results suggest that CBF-1 acts as a repressor for WC-independent *frq* transcription by promoting RCM-1 recruitment (Fig 4F–4H). WC-independent FRQ expression was previously observed in the *rco-1*^*KO*^ and *rcm-1*^*RIP*^ strains [27, 28, 46]. As shown here (Fig 1A and 1B and S3 Fig) and in a previous study [46], roles of CBF-1 and RCO-1/RCM-1 in control of the clock depend on conditions (i.e., glucose concentration and liquid/solid media). The absence of CBF-1 leads to long period or arrhythmic conidiation when glucose concentration is high (S3 Fig). The inconsistency in period of luciferase assay and race tube assay may also be caused by different media. Therefore, the absence of CBF-1 or RCO-1/RCM-1 leads to a more severe circadian phenotype in high glucose media.

In addition to the two functions of CBF-1 discussed above, the relatively low levels of FRQ protein but high *frq* mRNA level in the *cbf-1*^*KO*^ mutant suggest that CBF-1 has additional function in the clock. The inconsistency between *frq* RNA and FRQ protein was not unique for the *cbf-1* mutants and was previously also observed in *mcb* mutant [17]. In the *cbf-1* mutant, we showed that the WC-independent *frq* expression in the *cbf-1*mutant is sufficient to promote hyperphosphorylation of WC proteins and inhibit WCC DNA binding (Fig 4). Therefore, the relatively low FRQ level in the *cbf-1* mutant is sufficient to repress WCC activity. Comparison of the FRQ phosphorylation profiles showed that FRQ stayed constant hypophosphorylated in DD in different *cbf-1* mutants (Figs 1D and 4D), suggesting that CBF-1 can impact on FRQ phosphorylation due to an unknown mechanism. It is possible that these hypophosphorylated species of FRQ in the mutant is more potent for WCC inhibition than those in the wild-type strain. Consistent with this interpretation, a role for FRQ phopshorylation in the negative feedback loop was previously suggested by several studies [47, 48].

Here, we showed that the conserved transcription factor CBF-1, which contains HLH domain, plays an important role in the circadian negative feedback loop. CBF-1 is a member of the evolutionarily conserved bHLH-LZ transcription factor family [42]. Mammalian USF1, a homolog of *N*. *crassa* CBF-1, is a dominant suppressor of the *Clock*^*Δ19*^ mutation and competes with the CLOCK:BMAL1 complex for binding to E-box sites in target clock genes to regulate circadian gene expression [49]. The protein levels of CBF-1 in *N*. *crassa* or USF1 in mammals are very important for circadian clock. Therefore, our study here suggests that the regulation of positive element occupancy at the promoter of the negative element by CBF-1 homologues might be a conserved feature in eukaryotic circadian clock mechanisms. However, there are some differences in how CBF-1 and USF1 act in each clock system. CBF-1 suppresses *frq* transcription under normal conditions, whereas USF1 activates *per*/*cry* transcription when mutant CLOCK^Δ19^:BMAL1 is not transcriptional competent. In addition, E-box binding pattern of USF1 is antiphase to that of CLOCK. These differences might be evolutionary results from adaption to different clock systems.

## Materials and methods

### Strains, plasmid constructs, and growth conditions

The 87–3 (*bd*, *a*) strain was used as the wild-type strain in this study [50]. The *ku70*^*RIP*^ (*bd*, *a*) strain, generated previously [16], was used as the host strain for creating the *cbf-1* knockout mutant. The *cbf-1*^*KO*^ strain was created by deleting the entire *cbf-1* ORF through homologous recombination using a protocol described previously [51]. The *wc-1*^*KO*^, *wc-2*^*KO*^, *rcm-1*^*RIP*^ and *frq*^*9*^ strains, generated previously [28, 52–55], were also used in this study. The newly created *cbf-1*^*KO*^ *wc-2*^*KO*^ and *cbf-1*^*KO*^ *frq*^*9*^ double mutants were obtained by crossing. The 301–6 (*bd*, *his-3*, *A*) and *cbf-1*^*KO*^ (*bd*, *his-3*, *A*) strains were the host strains for *his-3* targeting construct transformation.

The *wt*, pqa-Myc-CBF-1, *wt*, pqa-Myc-CBF-1^E195A^, and *wt*, pqa-Myc-CBF-1^Δ(187–214)^ strains were created by transferring pqa-Myc-CBF-1, pqa-Myc-CBF-1^E195A^, and pqa-Myc-CBF-1^Δ(187–214)^ constructs into the *his-3* locus of 301–6 host strain. Using the same method, the *cbf-1*^*KO*^, pqa-Myc-CBF-1, *cbf-1*^*KO*^, pqa-Myc-CBF-1^E195A^, *cbf-1*^*KO*^, pqa-Myc-CBF-1^Δ(187–214)^, *cbf-1*^*KO*^, p*cbf-1*-FLAG-CBF-1, *cbf-1*^*KO*^, p*cbf-1*-FLAG-CBF-1^E195A^, and *cbf-1*^*KO*^, p*cbf-1*-FLAG-CBF-1^Δ(187–214)^ strains were generated. For each transformation, the transformants were first analyzed by western blot for the expression of tagged CBF-1 proteins, and the positive transformants were examined by race tube assays. *Escherichia coli* BL21 cells and pGEX-4T-1 plasmid were used for expression of GST-CBF-1 and GST-CBF-1^Δ(187–214)^ fusion proteins.

The medium for race tube assays contained 1x Vogel’s salts, 0.1% glucose, 0.17% arginine, 50 ng/mL biotin, and 1.5% agar. In the race tube medium containing QA, 0.1% glucose was replaced with the desired concentration of QA (0-10^-2^ M). Strains were grown in constant light at 25°C for 24 hours before being transferred to DD at 25°C. Densitometric analyses of race tubes and calculations of period length were performed as described [56]. Growth conditions were as described previously [57]. Liquid cultures were grown in minimal medium (1x Vogel’s, 2% glucose). When QA was used, liquid cultures were grown in low-glucose medium (1x Vogel’s, 0.1% glucose, 0.17% arginine) with different concentration of QA (0-10^-2^ M).

### Luciferase reporter assays

The luciferase reporter assays were performed as described previously [58, 59]. The 301–6 (*bd*, *A*), *frq*-*luc* strain was used as control strain in this study. The *cbf-1*^*KO*^ strains were crossed with the 301–6 (*bd*, *A*), *frq*-*luc* strain to obtain the *cbf-1*^*KO*^, *frq-luc* strain. The luciferase reporter construct was co-transformed with a pBT6 plasmid into CBF-1 overexpression strain to obtain *wt*, qa-Myc-CBF-1, *frq-luc* strain. LumiCycle (ACTIMETRICS) and the autoclaved fructose-glucose-sucrose (FGS)-Vogel’s medium (1x FGS, 1x Vogel’s medium, 50 μg/L biotin, and 1.8% agar) containing 50 μM firefly D-luciferin were used for the luciferase assay. Conidia suspensions in water were placed on autoclaved FGS-Vogel’s medium and grown in constant light overnight. The cultures were then transferred to constant darkness, and luminescence was recorded in real time using a LumiCycle after one day in DD. The data were normalized with LumiCycle Analysis Software by subtracting the baseline luciferase signal which increases as cell grows. Under our experimental conditions, luciferase signals are highly variable during the first day in the LumiCycle and become stabilized afterwards, which is likely due to an artifact caused by the light-dark transfer of the cultures. Thus, the results presented were recorded after one day in DD.

### Generation of antiserum against CBF-1, WC-1, WC-2, RCM-1 and FRQ

The GST-CBF-1 (amino acids M1-N136), GST-WC-1 (amino acids G291-E708), GST-WC-2 (amino acids M8-C423), GST-RCM-1 (amino acids D480-P883) [28], and GST-FRQ (amino acids S249-K315 and E359-G766) [60] fusion proteins were expressed in *E*. *coli* BL21 cells, and the recombinant proteins were purified and used as the antigens to generate rabbit polyclonal antiserum as described previously [61].

### Protein and RNA analyses

Protein extraction, quantification, western blot analyses, and co-immunoprecipitation assays were performed as previously described [62]. Equal amounts of total protein (40 μg) were loaded in each protein lane. After electrophoresis, proteins were transferred onto PVDF membrane, and western blot analysis was performed. To analyze the phosphorylation profiles of WC-1, WC-2, and RCM-1, phosphatase inhibitors were added to protein extraction buffer and 7.5% SDS-PAGE gels containing a ratio of 149:1 acrylamide/bis-acrylamide were used. Otherwise, 7.5% SDS-PAGE gels contained a ratio of 37.5:1 acrylamide/bis-acrylamide were employed.

Total RNA was extracted using Trizol, and then further purified with 2.5 M LiCl as described previously [63]. For northern blot analysis, equal amounts of total RNA (20 μg) were loaded onto agarose gels. After electrophoresis, the RNA was transferred onto Amersham Hybond-N+ membrane. The membrane was probed with ^32^P-UTP-labeled RNA probes specific for *frq*, *wc-1*, or *wc-2*. RNA probes were transcribed *in vitro* from PCR products by T7 RNA polymerase. The northern primer sequences used for the template amplification were *frq*-N term F (5’-GGGTAGTCGTGTACTTTGTCAGGCATAGATCTC-3’), *frq*-N-term T7 + R (5’-TAATACGACTCACTATAGGGGGCAGGGTTACGATTGGATT-3’), *wc1* F (5’-GTTATACCTGGTTTGAAAGC-3’), *wc1* T7 + R (5’-TAATACGACTCACTATAGGGACAACTGTTGCATAGATCTC-3’), *wc2* F (5’-CTGCAGATGACTTCCGACCC-3’), and *wc2* T7 + R (5’-TAATACGACTCACTATAGGGATCTCGGTCTAGGGGAATC-3’). The T7 promoter regions are underlined.

### EMSA assays

EMSA assays were performed in a manner similar to that described previously [64]. To make the probe, oligonucleotides were dissolved in double distilled H_2_O (ddH_2_O) to a final concentration of 10 μM. We then mixed 10 μL each of complementary oligonucleotides with 30 μL ddH_2_O and heated at 95°C for 10 minutes. After overnight at room temperature, oligonucleotide labeling was performed at 37°C for 30 minutes in 50 μL reaction with 5 μL of double-stranded oligonucleotide, 5 μL of T4 polynucleotide kinase buffer (NEB), 2.5 μL of T4 polynucleotide kinase (NEB), 7.5 μCi γ^32^P-ATP and 30 μL ddH_2_O. After the kinase reaction, the sample was purified using Bio-Gel P-30 chromatography columns (Bio-Rad). The oligonucleotides annealed for use as probe were ACF57 (5’-CGTCCTGATGCCGCTGCAAGACCGATGACGCTGCAAAATTGAGATCTA-3’); and ACF58 (5’-TAGATCTCAATTTTGCAGCGTCATCGGTCTTGCAGCGGCATCAGGACG-3’).

Binding reactions using BL21 cells expressing fusion protein contained 1x binding buffer [20 mM HEPES, pH 7.9, 1 mM EDTA, 2 mM MgCl_2_, 10% (v/v) glycerol, 20 μM ZnCl_2_], 0.1 μg poly (dI-dC), 1 μL probe, and 3 μg of GST, GST-CBF-1, or GST-CBF-1^Δ(187–214)^ fusion protein (which was added last to the binding reactions) in a total volume of 20 μL. Binding reactions were incubated for 30 minutes on ice prior to electrophoresis at 4°C on nondenaturing 4% polyacrylamide gels containing 0.5x TBE and 2.5% (v/v) glycerol. Gels were dried at 80°C for 15 minutes and were exposed it to X-ray film for 2 to 10 hours.

### ChIP analyses

ChIP assays were performed as described previously [62]. Briefly, *N*. *crassa* tissues were fixed with 1% formaldehyde for 15 minutes at 25°C with shaking. Glycine was added at a final concentration of 125 mM, and samples were incubated for another 5 minutes. The crosslinked tissues are ground and resuspended at 0.5 g in 6 mL lysis buffer containing protease inhibitors (1 mM PMSF, 1 μg/mL leupeptin and 1 μg/mL pepstatin A). Chromatin was sheared by sonication to approximately 200–500 base pair fragments. A 1mL aliquot of protein solution (2 mg/mL) was used for each immunoprecipitation reaction, and 10 μL was kept as the input DNA. The chromatin immunoprecipitations were carried out with 2.5 μL WC-2, 3.5 μL WC-1, 2.5 μL RCM-1, or 3 μL CBF-1 antibodies. The corresponding knock-out strains were used as the negative controls. Immunoprecipitated DNA was quantified using real-time PCR. The primer sets used are *frq* C-box F (5’-GTCAAGCTCGTACCCACATC-3’) and *frq* C-box R (5’-CCGAAAGTATCTTGAGCCTCC-3’) were described in a previous study [29]. Occupancies were normalized by the ratio of ChIP to Input. The relative values of protein occupancy were calculated using the 2^-ΔΔCT^ method by comparing the cycle number for ChIP sample with that for the Input control [65].

### Quantifications and statistical analyses

Quantification of western blot and northern blot data were performed using Quantity One software. All experiments were performed at least three independent times. For blots, representative images are shown. Error bars are standard deviations of triplicate data. Statistical significance was determined by Student’s t test for ChIP analyses.

## Supporting information

S1 Fig FRQ stability is not affected in the *cbf-1*^*KO*^ strain. Western blot analyses showing the relative levels of FRQ protein after addition of 10 μg/ml cycloheximide (CHX) in the wild-type and *cbf-1*^*KO*^ strains.
(TIF)

S2 Fig Expression of Myc-CBF-1 in *cbf-1*^*KO*^, qa-Myc-CBF-1, and *wt*, qa-Myc-CBF-1 strains. (A) Western blot analyses of the levels of Myc-CBF-1 in *cbf-1*^*KO*^, qa-Myc-CBF-1 strain with different QA concentrations (0 to 10^−2^ M). (B) Western blot analyses of the levels of Myc-CBF-1 in *wt*, qa-Myc-CBF-1 strain with different QA concentrations (0 to 10^−2^ M). (C) Western blot analyses of the levels CBF-1 and Myc-CBF-1 in *wt*, qa-Myc-CBF-1 strains with 10^−3^ M QA.
(TIF)

S3 Fig Circadian conidiation rhythm is not affected by glucose concentration in the *cbf-1*^*KO*^, qa-Myc-CBF-1 strain. The conidiation rhythm of wild-type, *cbf-1*^*KO*^ and *cbf-1*^*KO*^, qa-Myc-CBF-1 strains are shown on race tubes containing different concentrations of glucose.
(TIF)

S4 Fig Overexpressed CBF-1 negatively regulates levels of WC-1 and WC-2 proteins in a post-transcriptional manner. (A) Western blot analyses of WC-1 and WC-2 protein levels in the wild-type and CBF-1 overexpressing strains. (B) Northern blot analyses of the levels of *wc-1* and *wc-2* mRNA in the wild-type and CBF-1 overexpressing strains.
(TIF)

S5 Fig Expression of GST-CBF-1 fusion protein and the protein levels of WCC in the *cbf-1*^*KO*^ strain. (A) The expression of GST-CBF-1-related fusion proteins analyzed by staining of an SDS-PAGE gel with Coomassie blue. (B) Western blot analyses of WC-1 and WC-2 protein levels in the wild-type and *cbf-1*^*KO*^ strains.
(TIF)

S6 Fig The protein levels of WCC are similar between *frq*^*9*^ single mutant and *cbf-1*^*KO*^ *frq*^*9*^ double mutant strains. (A) Western blot analyses of the levels of CBF-1 and FRQ proteins in the wild-type, *wc-2*^*KO*^, *frq*^*9*^, *cbf-1*^*KO*^, and *cbf-1*^*KO*^ *frq*^*9*^ strains. (B) Western blot analyses of the levels of WC-1 and WC-2 proteins in the wild-type, *wc-2*^*KO*^, *frq*^*9*^, *cbf-1*^*KO*^, and *cbf-1*^*KO*^ *frq*^*9*^ strains.
(TIF)

S7 Fig The protein levels of WCC are not affected in the CBF-1 DNA binding defect mutants. (A) Western blot analyses of the levels of CBF-1 and FLAG-CBF-1 in CBF-1 DNA binding defect mutants. (B) Western blot analyses and quantification of the levels of WC-1 in CBF-1 DNA binding defect mutants. (C) Western blot analyses and quantification of the levels of WC-2 protein in CBF-1 DNA binding defect mutants.
(TIF)

S8 Fig The levels of WCC and FRQ are similar in the wild-type and CBF-1 overexpression strains. Western blot analyses of the levels of CBF-1, WC-1, WC-2 and FRQ proteins in the wild-type and CBF-1 overexpression strains.
(TIF)
