# Supplementary figures and images for: Transcription factor CBF-1 is critical for circadian gene expression by modulating WHITE COLLAR complex recruitment to the frq locus
Source: PLoS Genet. 2018 Sep 12;14(9):e1007570. doi: 10.1371/journal.pgen.1007570 (PMC6152987; doi:10.1371/journal.pgen.1007570)

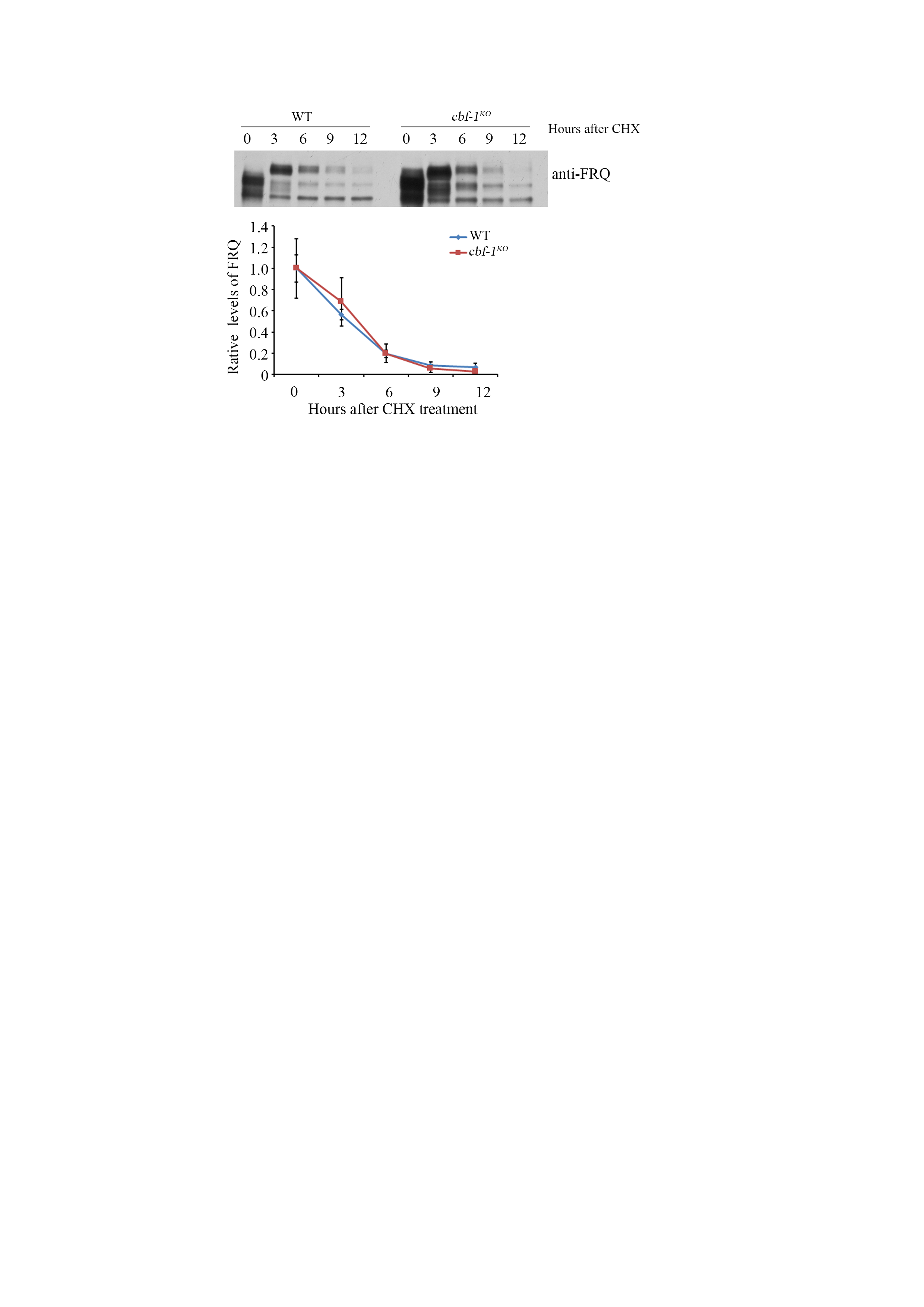

Supplement: S1 Fig — Western blot analyses showing the relative levels of FRQ protein after addition of 10 μg/ml cycloheximide (CHX) in the wild-type and cbf-1KO strains. (TIF) [file pgen.1007570.s001.tif]

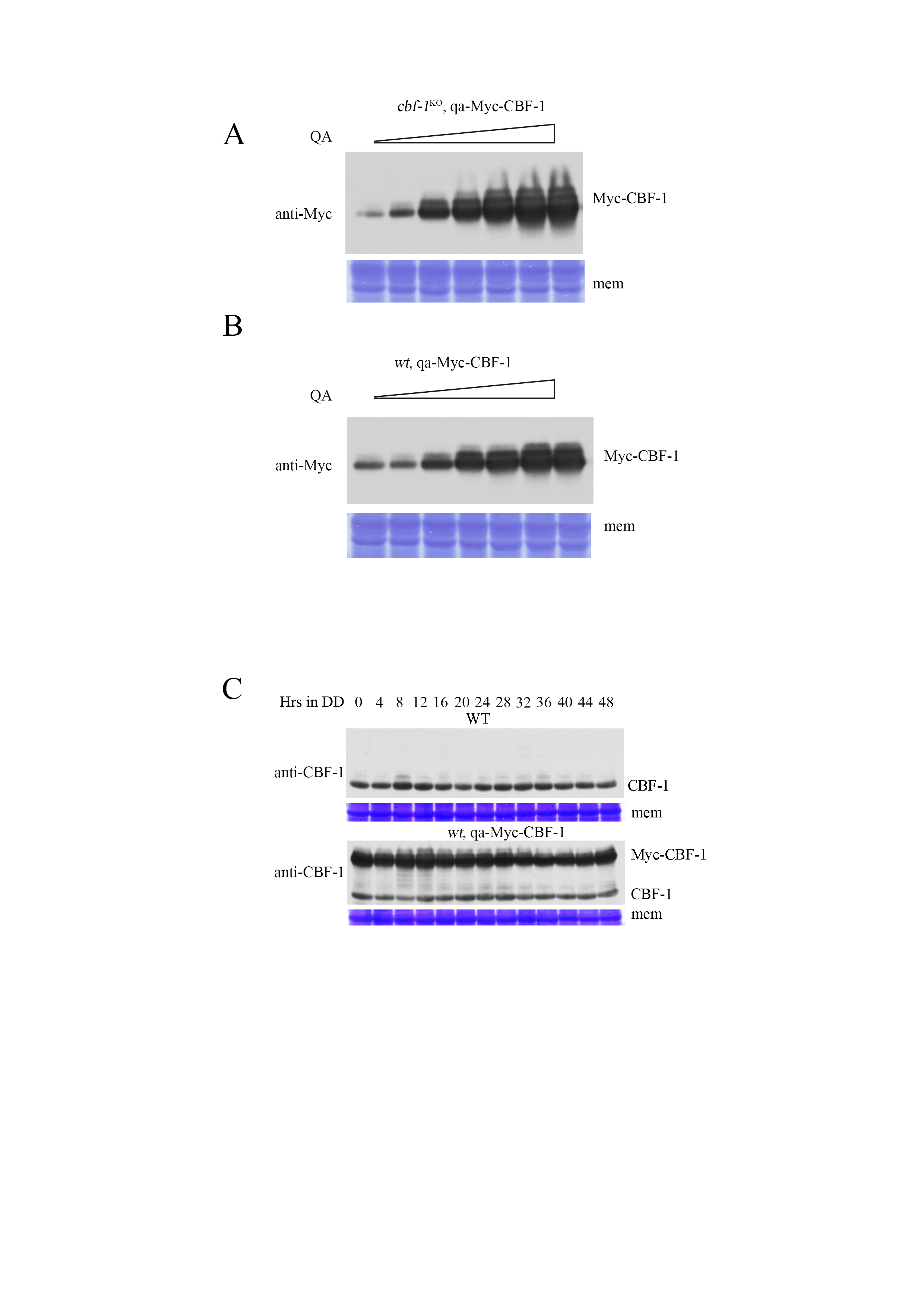

Supplement: S2 Fig — (A) Western blot analyses of the levels of Myc-CBF-1 in cbf-1KO, qa-Myc-CBF-1 strain with different QA concentrations (0 to 10−2 M). (B) Western blot analyses of the levels of Myc-CBF-1 in wt, qa-Myc-CBF-1 strain with different QA concentrations (0 to 10−2 M). (C) Western blot analyses of the levels CBF-1 and Myc-CBF-1 in wt, qa-Myc-CBF-1 strains with 10−3 M QA. (TIF) [file pgen.1007570.s002.tif]

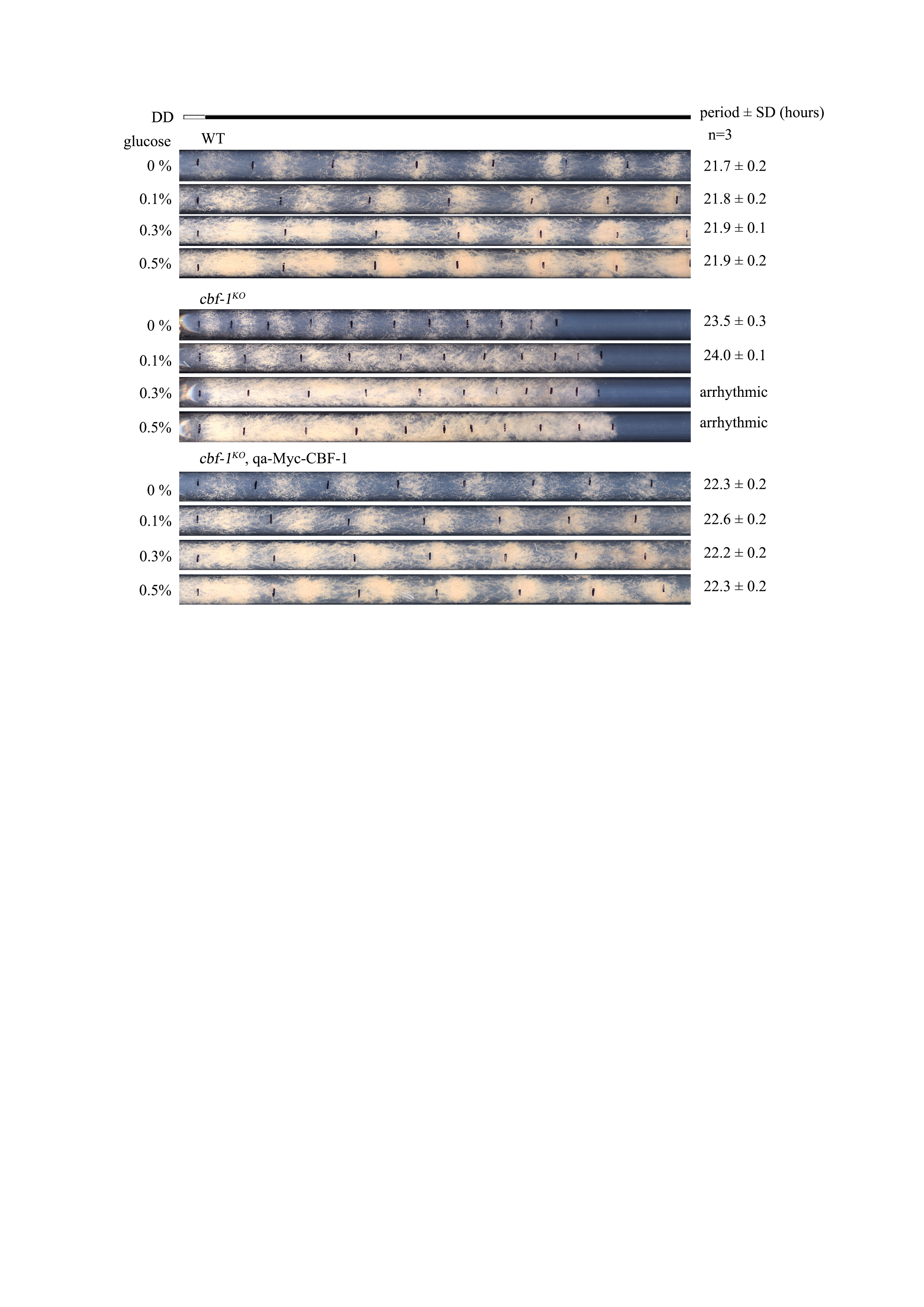

Supplement: S3 Fig — The conidiation rhythm of wild-type, cbf-1KO and cbf-1KO, qa-Myc-CBF-1 strains are shown on race tubes containing different concentrations of glucose. (TIF) [file pgen.1007570.s003.tif]

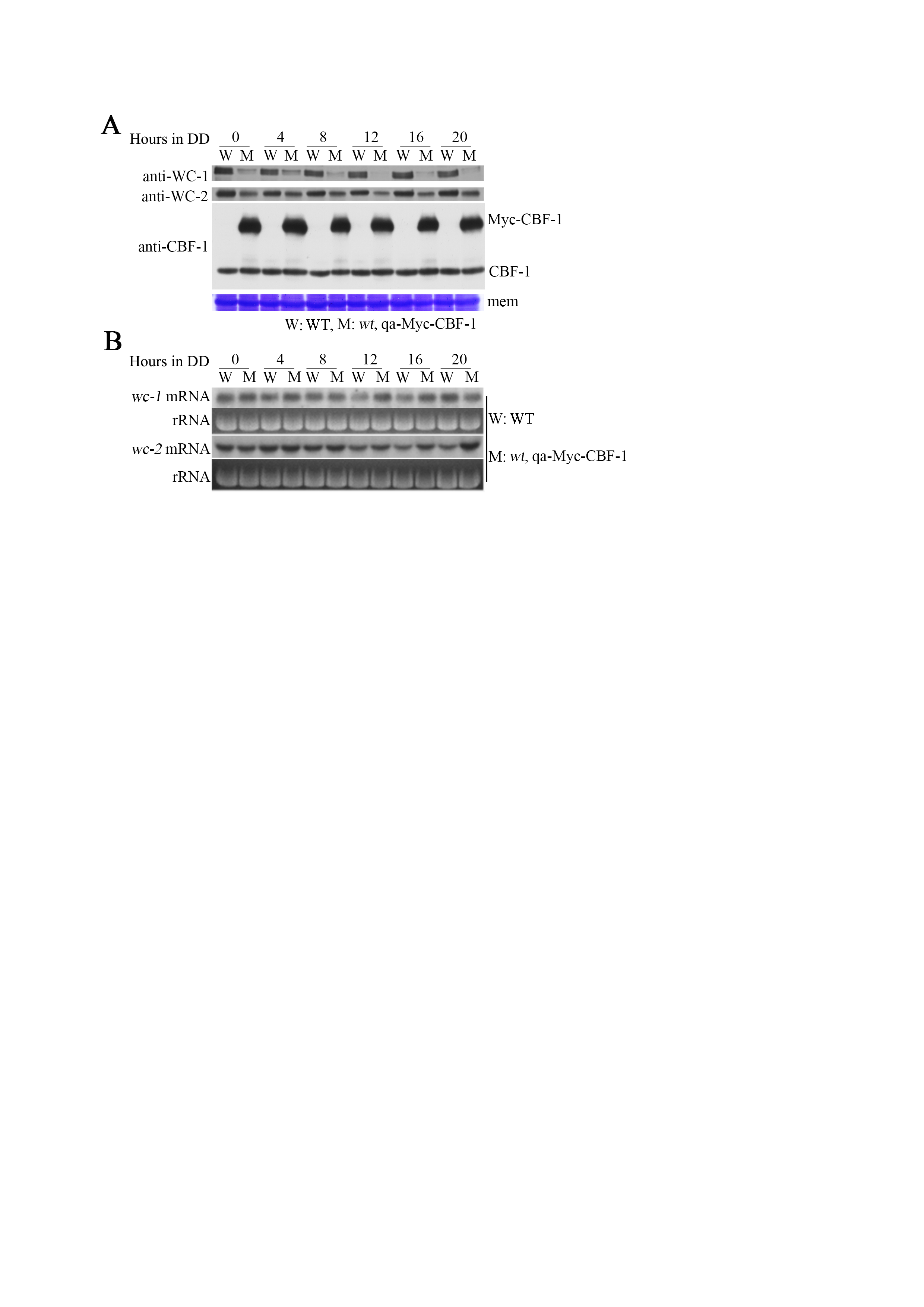

Supplement: S4 Fig — (A) Western blot analyses of WC-1 and WC-2 protein levels in the wild-type and CBF-1 overexpressing strains. (B) Northern blot analyses of the levels of wc-1 and wc-2 mRNA in the wild-type and CBF-1 overexpressing strains. (TIF) [file pgen.1007570.s004.tif]

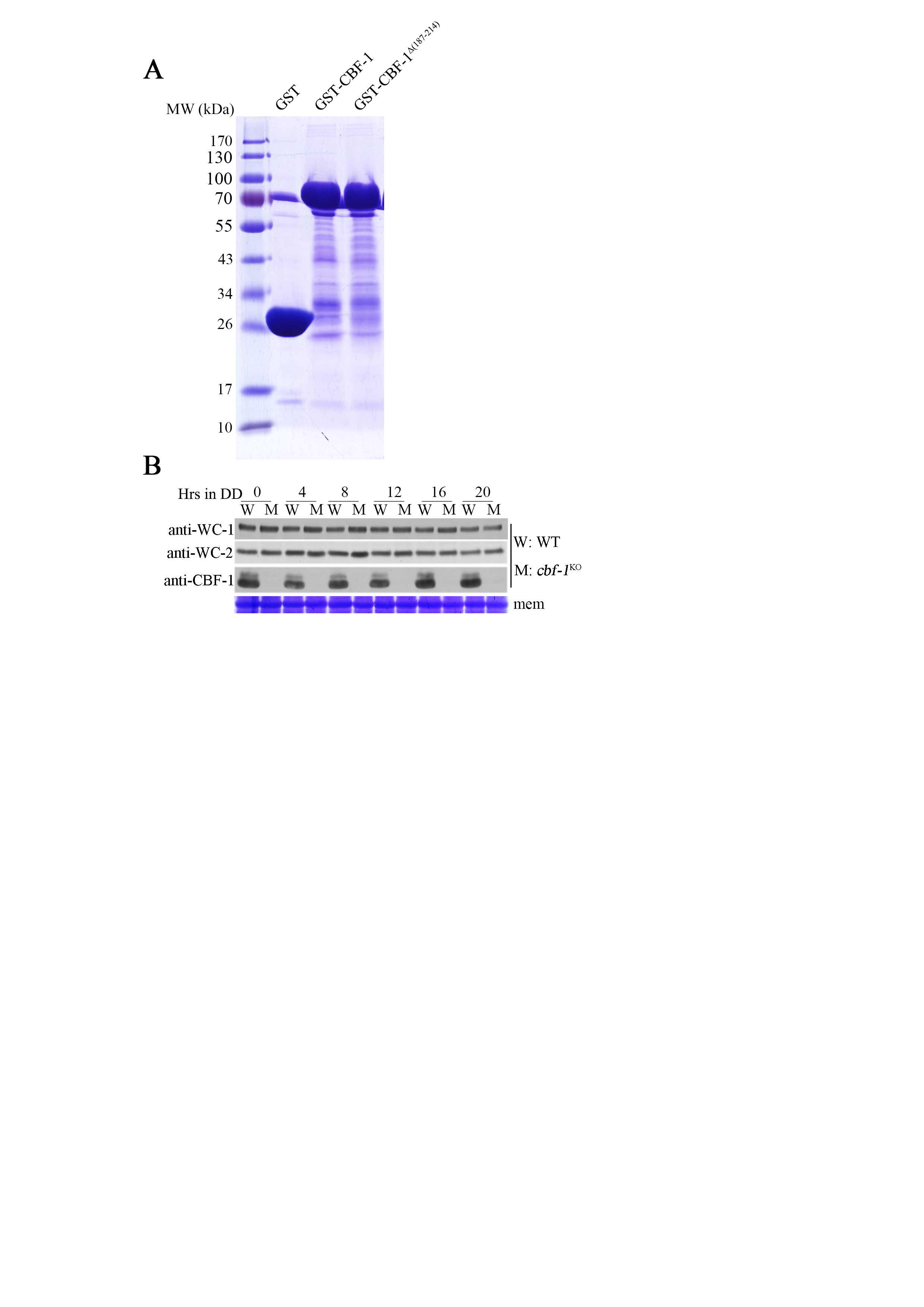

Supplement: S5 Fig — (A) The expression of GST-CBF-1-related fusion proteins analyzed by staining of an SDS-PAGE gel with Coomassie blue. (B) Western blot analyses of WC-1 and WC-2 protein levels in the wild-type and cbf-1KO strains. (TIF) [file pgen.1007570.s005.tif]

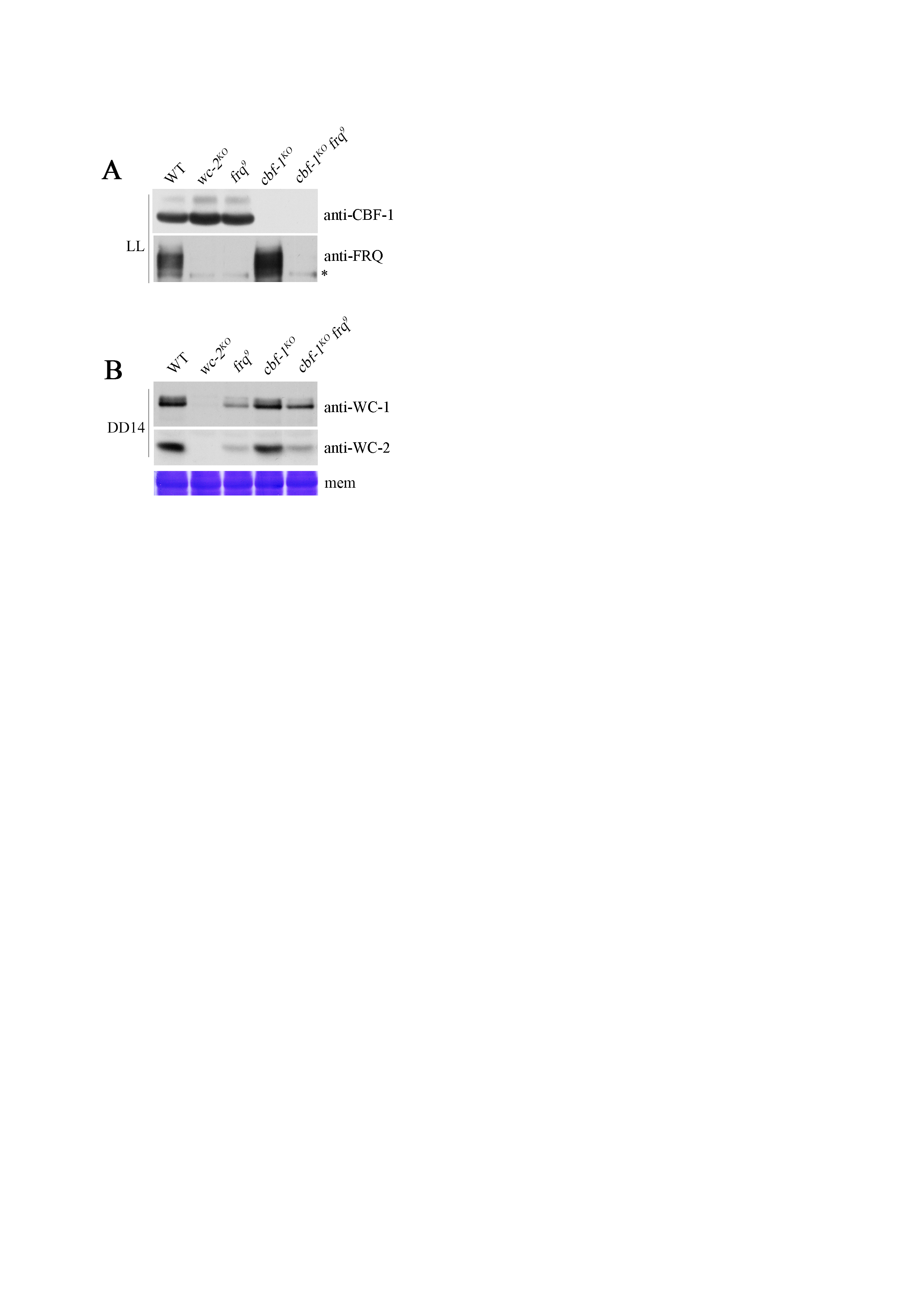

Supplement: S6 Fig — (A) Western blot analyses of the levels of CBF-1 and FRQ proteins in the wild-type, wc-2KO, frq9, cbf-1KO, and cbf-1KO frq9 strains. (B) Western blot analyses of the levels of WC-1 and WC-2 proteins in the wild-type, wc-2KO, frq9, cbf-1KO, and cbf-1KO frq9 strains. (TIF) [file pgen.1007570.s006.tif]

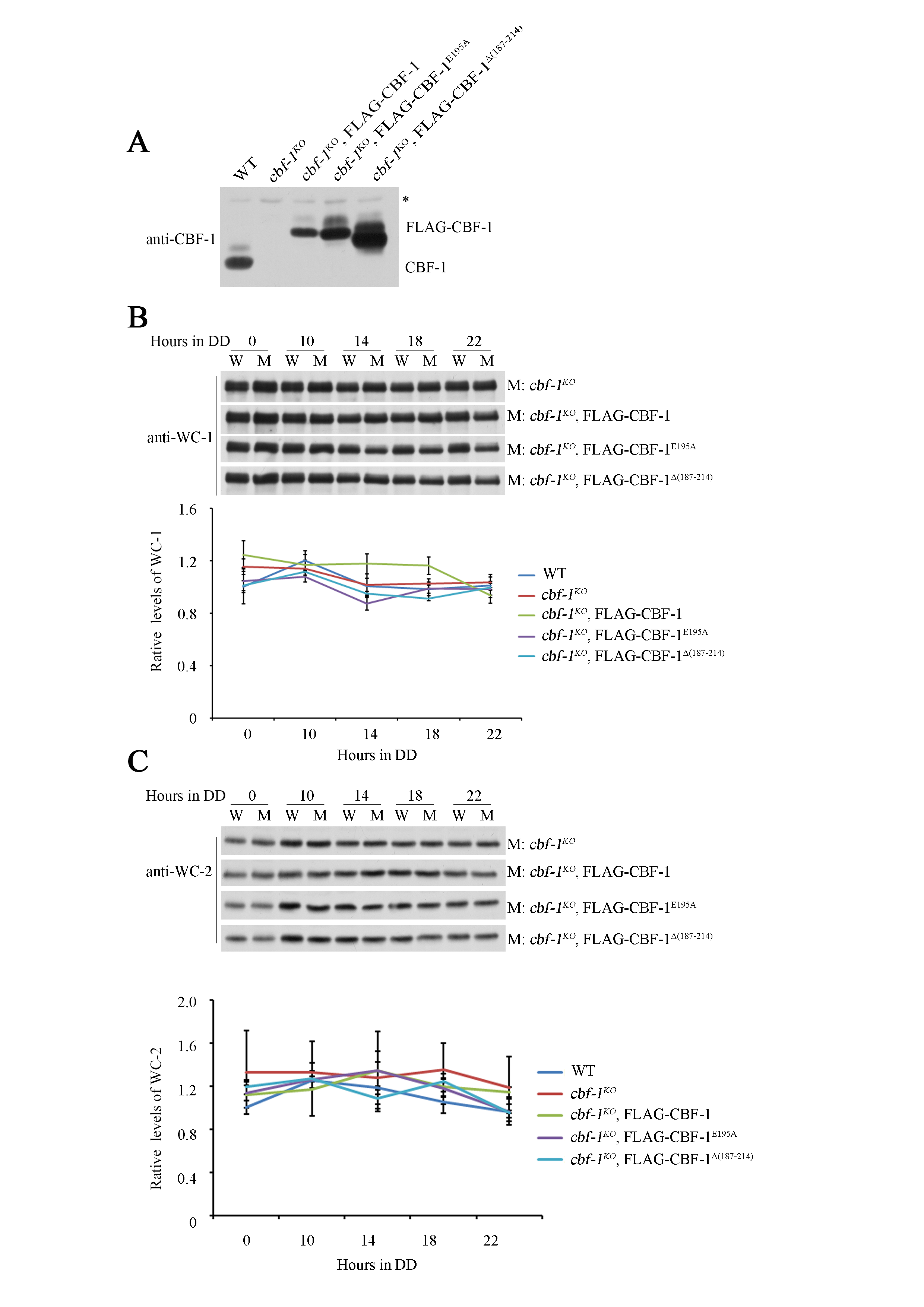

Supplement: S7 Fig — (A) Western blot analyses of the levels of CBF-1 and FLAG-CBF-1 in CBF-1 DNA binding defect mutants. (B) Western blot analyses and quantification of the levels of WC-1 in CBF-1 DNA binding defect mutants. (C) Western blot analyses and quantification of the levels of WC-2 protein in CBF-1 DNA binding defect mutants. (TIF) [file pgen.1007570.s007.tif]

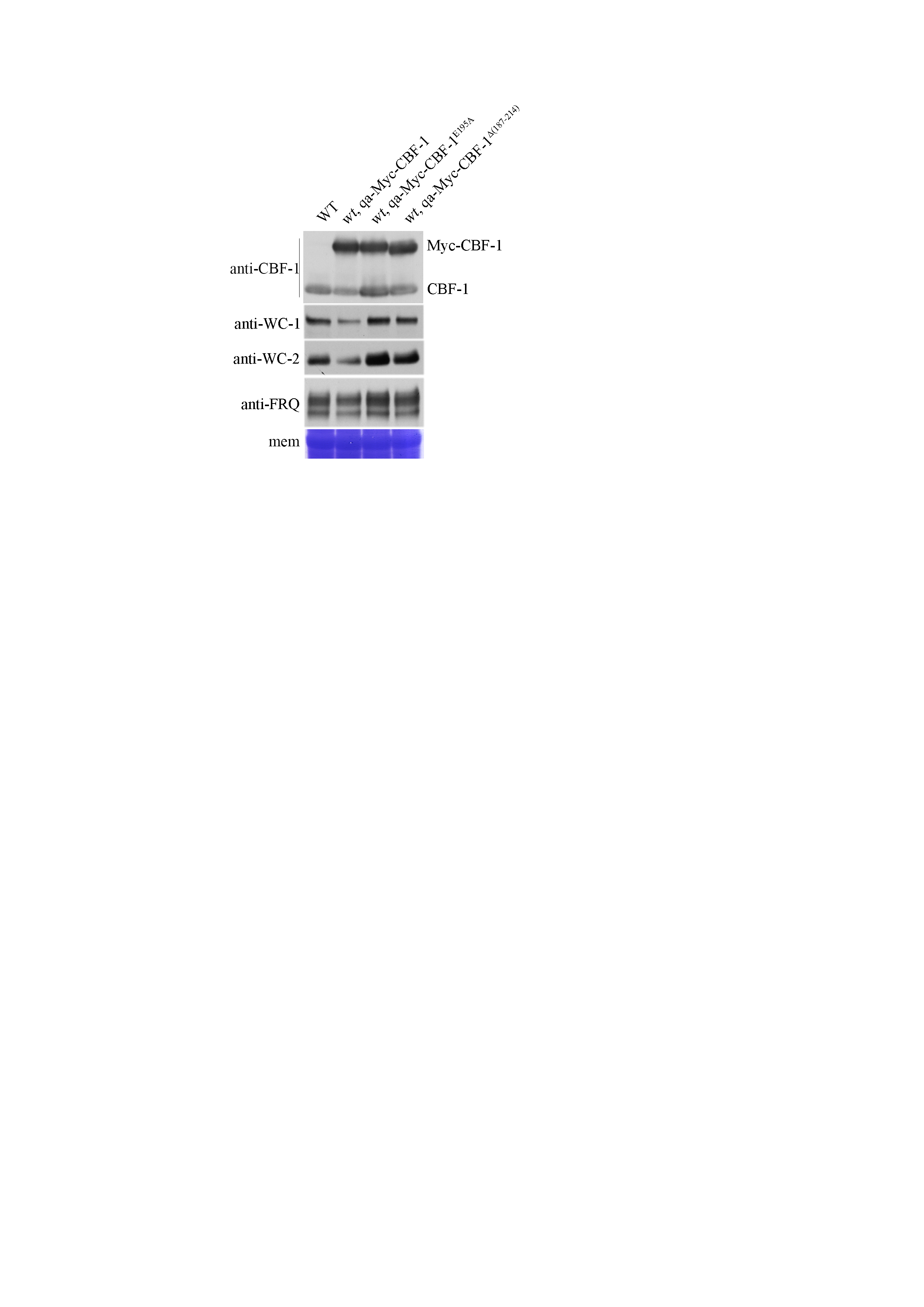

Supplement: S8 Fig — Western blot analyses of the levels of CBF-1, WC-1, WC-2 and FRQ proteins in the wild-type and CBF-1 overexpression strains. (TIF) [file pgen.1007570.s008.tif]
